# Continuous Condensed Triplet Accumulation for Irradiance‐Induced Anticounterfeit Afterglow

**DOI:** 10.1002/advs.202304374

**Published:** 2023-10-28

**Authors:** Ende Hopsah Badriyah, Kikuya Hayashi, Bahadur Sk, Rina Takano, Takayuki Ishida, Shuzo Hirata

**Affiliations:** ^1^ Department of Engineering Science The University of Electro‐Communications 1‐5‐1 Chofugaoka Chofu Tokyo 182–8585 Japan

**Keywords:** afterglow emission, Förster resonance energy transfer, high‐resolution microscopy, room‐temperature phosphorescence, triplet exciton

## Abstract

Afterglow room‐temperature emission that is independent of autofluorescence after ceasing excitation is a promising technology for state‐of‐the‐art bioimaging and security devices. However, the low brightness of the afterglow emission is a current limitation for using such materials in a variety of applications. Herein, the continuous formation of condensed triplet excitons for brighter afterglow room‐temperature phosphorescence is reported. (*S*)‐(‐)‐2,2′‐Bis(diphenylphosphino)‐1,1′‐binaphthyl ((*S*)‐BINAP) incorporated in a crystalline host lattice showed bright green afterglow room‐temperature phosphorescence under strong excitation. The small triplet–triplet absorption cross‐section of (*S*)‐BINAP in the whole range of visible wavelengths greatly suppressed the deactivation caused by Förster resonance energy transfer from excited states of (*S*)‐BINAP to the accumulated triplet excitons of (*S*)‐BINAP under strong continuous excitation. The steady–state concentration of the triplet excitons for (*S*)‐BINAP reached 2.3 × 10^−2^ M, producing a bright afterglow. Owing to the brighter afterglow, afterglow detection using individual particles with sizes approaching the diffraction limit in aqueous conditions and irradiance‐dependent anticounterfeiting can be achieved.

## Graphical abstract

Continuous triplet accumulation over 1 wt.% in a host‐guest crystalline material showing bright green afterglow emission is achieved due to suppressed Förster resonance energy transfer for accumulated triplet excitons under strong excitation. The medium, as well as a common green afterglow emitter, exhibits a promising irradiance‐induced appearance for high‐resolution afterglow imaging, providing novel anticounterfeiting capabilities.

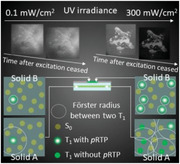

## 1 Introduction

Afterglow room‐temperature (RT) emission that persists after ceasing excitation in ambient conditions enables emission imaging that is independent of autofluorescence from background impurities.^[^ ^1^, ^2^, ^3^, ^4^, ^5^, ^6^, ^7^ ^]^ Because afterglow RT emission for more than 100 ms after excitation can be detected without a large decrease of the emission using cost‐effective and portable 2D photodetectors,^[^ ^8^ ^]^ materials showing RT afterglow can be used for state‐of‐the‐art bioimaging^[^ ^3^, ^9^, ^10^, ^11^, ^12^, ^13^, ^14^, ^15^, ^16^, ^17^ ^]^ and security imaging.^[^ ^18^, ^19^, ^20^, ^21^, ^22^, ^23^ ^]^ However, the brightness of the afterglow RT emission is commonly weak compared with that of fluorescence, which becomes a bottleneck for using the materials in a variety of other applications. Compared with materials showing persistent delayed emission after charge recombination,^[^ ^24^, ^25^ ^]^ persistent RT phosphorescence (RTP) has a rapid radiation but hardly decreases emission intensity for 100 ms after excitation ceases. Therefore, the emission observed for 100 ms to 1 s after ceasing excitation from materials with persistent RTP is brighter compared with the persistent delayed emission materials.^[^ ^26^ ^]^

To obtain a higher brightness of persistent RTP, improving the quantum yield (Φ_p_) corresponding to photons lasting over 100 ms is crucial. Although the increase of Φ_p_ is important for energy conversion applications, the detection of persistent RTP from small‐scale materials is necessary for applications with high resolution. For the detection of the afterglow signal from small‐scale materials, sufficient Φ_p_ under strong excitation intensity is required because the increase of excitation intensity is mandatory for high‐resolution imaging applications. However, a low Φ_p_ in strong excitation has always been observed for previously reported persistent RTP materials.^[^ ^27^, ^28^ ^]^ The saturation of the triplet exciton concentration in the materials is considered a potential reason for the large decrease of Φ_p_ under strong excitation.^[^ ^25^, ^26^ ^]^ Therefore, approaches must be developed to increase the triplet concentration in the materials and increase the brightness of afterglow RT emissions.

Herein, we report continuous accumulation of RT triplet excitons over 1 wt.% and enhanced irradiance‐independent anticounterfeit phenomena. Organic green phosphorescent fluorene derivatives doped into amorphous *β*‐estradiol was prepared as an amorphous solid, and (*S*)‐(‐)−2,2′‐bis(diphenylphosphino)−1,1′‐binaphthyl ((*S*)‐BINAP) incorporated in a crystalline lattice of (*S*)‐(–)−2,2′‐bis(diphenylphosphino)−5,5′,6,6′,7,7′8,8′‐octahydro‐1,1′‐binaphthyl ((*S*)‐H_8_‐BINAP) was prepared as a crystalline material. Thin films of the two materials showed comparable persistent RTP brightness under weak excitation because of comparable triplet generation capability and Φ_p_. However, the (*S*)‐BINAP‐doped crystalline film showed ≈10 times larger brightness compared with the fluorene derivative‐doped amorphous film under strong excitation. This allowed for no afterglow images under weak excitation but selective high‐resolution afterglow images under strong excitation, which can be exploited in state‐of‐the‐art anticounterfeiting applications. For previously reported materials, including the fluorene derivative‐doped film, the largest magnitudes of the continuous concentration of RT triplet excitons have been on the order of 10^−3^ M. However, the (*S*)‐BINAP‐doped crystalline film showed continuous concentrations of RT triplet excitons up to 2.3 × 10^−2^ M. Although significant exciton quenching caused by Förster resonance energy transfer (FRET) from singlet and triplet states precluded the triplet accumulation, a small triplet–triplet (T–T) absorption cross‐section of (*S*)‐BINAP over the visible wavelengths largely suppressed the exciton quenching caused by FRET, allowing the continuous condensed triplet accumulation under strong excitation. An afterglow image was observed from individual particles of the (*S*)‐BINAP‐doped (*S*)‐H_8_‐BINAP crystals with sizes approaching the diffraction limit in ambient aqueous conditions.

## 2 Results and Discussion

### 2.1 Excitation Irradiance‐Independent High‐Resolution Anticounterfeiting

**Figure 1:**
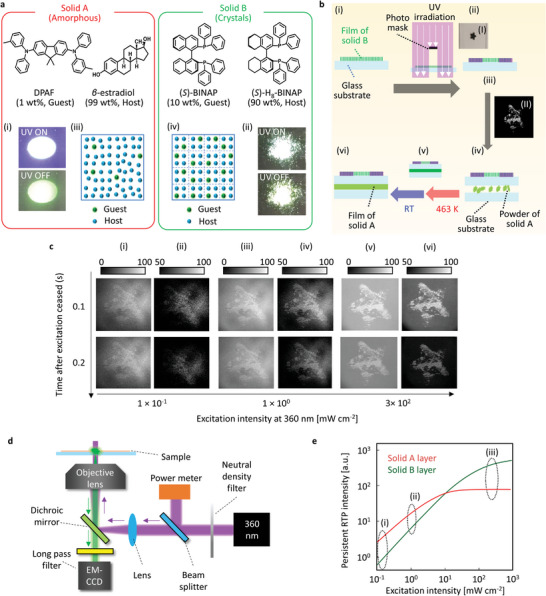
The irradiance‐dependent appearance of afterglow images. a) Two kinds of molecular solid (Solids A and B). Green afterglow emission of the solid A (i) and solid B (ii) after excitation at 360 nm ceased. Illustrations to explain the condition of guest chromophores in the host for solid A (iii) and solid B (iv). The color photographs were taken with a Ricoh R8 camera. b) Preparation procedures (i)‐(vi) of sample for irradiance‐dependent appearance of afterglow images. Inset photographs (I) and (II) represent a photomask and afterglow RT emission from the sample in the procedure (ii) after ceasing excitation at 360 nm and 10 mW cm^−2^ in air. c) Irradiance‐dependent appearance of high‐resolution afterglow images. The excitation wavelength is 360 nm. Monochrome images were measured with the optical setup shown in (d), and the excitation light power was varied with the use of a neutral‐density filter. d) Epi‐emission microscopic setup used to view the irradiance‐dependent appearance of high‐resolution afterglow images. e) Illustration to explain different characteristics of persistent RT emission intensity change of solids A and B depending on excitation intensity.

First, 1 wt.% 9,9‐dimethyl‐*N* ^2^,*N* ^7^‐diphenyl‐*N* ^2^,*N* ^7^‐di‐*m*‐tolyl‐9*H*‐fluorene‐2,7‐diamine (DPAF) doped amorphous *β*‐estradiol film (solid A)^[^ ^4^ ^]^ and 10 wt.% (*S*)‐BINAP‐doped (*S*)‐H_8_‐BINAP single crystals (solid B) were prepared (**Figure** **1a**, see Section S1 in Supporting Information for more details). The solids A and B generate green afterglow RT emission for a short time after excitation at 360 nm ceased (Figure 1a (i) and (ii), respectively). In solid A, DPAF is molecularly dispersed in amorphous *β*‐estradiol (Figure 1a(iii)).^[^ ^4^ ^]^ In solid B, cooperative analysis of single crystalline X‐ray and nuclear magnetic resonance measurements indicates that (*S*)‐H_8_‐BINAP in a crystalline lattice of (*S*)‐H_8_‐BINAP host is randomly replaced by (*S*)‐BINAP because of their similar molecular size and shape (Figure 1a (iv); Figures S1–S3 and Table S1, Supporting Information), which have been reported for different concentrations of (*S*)‐BINAP in (*S*)‐H_8_‐BINAP crystal.^[^ ^29^ ^]^ An anticounterfeit medium was fabricated using solids A and B in the following procedure. A polycrystalline film of solid B was prepared on a glass substrate (Figure 1b(i), Section 1, Supporting Information). The film was irradiated with UV light using a wavelength of 360 nm and power of 1 W cm^−2^ for 3 min through a photomask (Figure 1b, (ii)). Soon after a weak (10 mW cm^−2^) UV light (360 nm) is applied to the solid B film, an afterglow pattern with the shape of a star from the solid B was observed because of photobreaching according to the shadow pattern of the photomask (Figure 1b (iii)). Next, the powder of solid A was sandwiched between the side of the glass substrate with the photopatterned solid B film and another glass substrate (Figure 1b (iv)). By heating the sandwiched sample at 190 °C, solid A melted and spread out between two glass substrates via capillary action (Figure 1b, (v)), and an anticounterfeit medium was obtained by quenching the sample at RT (Figure 1b, (vi)).

When the anticounterfeit medium is set under an epi‐fluorescence microscope and excitation at 360 nm was applied to the medium with the power of 0.1 mW cm^−2^, the star‐shaped afterglow pattern of the solid B layer extinguished soon after ceasing the excitation (Figure 1c (i), Figure 1d, Movie S1, Supporting Information) because the green afterglow emission intensity from the solid B layer is less than that from the solid A layer (Figure 1e (i)). The star‐shaped afterglow from the solid B layer did not appear clearly, even when the top 50% brightness was graded from white to black (Figure 1c (ii)). The afterglow RTP intensity of the solid B layer increased more compared with the solid A when the excitation intensity at 360 nm increased from 0.1 to 1.0 mW cm^−2^ (Figure 1e (ii)). The star‐shaped location of the solid B layer could be distinguished slightly more easily using higher power excitation (Figure 1c (iii) and (iv)). When the excitation intensity at 360 nm increases to 300 mW cm^−2^, the afterglow RTP intensity of the solid B layer significantly increased while the increase in the RTP intensity of the solid A layer was relatively small (Figure 1e (iii)). Therefore, the star shape of afterglow emission from the solid B layer was observed more clearly (Figure 1c (v), Movie S2, Supporting Information), and the star‐shaped afterglow emission was clearer when the top 50% brightness was graded from white to black (Figure 1c (vi)). The star‐shaped afterglow that appeared selectively under strong excitation is not caused by photodegradation of the solid A because the star‐shaped afterglow image does not appear when the weak excitation power (0.1 mW cm^−2^) is used again. The distinguishable irradiance‐dependent afterglow was also observed when single crystals of solid B were placed on the powder sample of solid A (Figure S4, Supporting Information). Notably, a large contrast in the emission images based on continuous excitation has not been previously reported. This type of irradiance‐induced high‐resolution emission imaging provides novel anticounterfeiting capabilities.

### 2.2 Linear Photophysical Properties of Materials Under Weak Excitation

**Figure 2:**
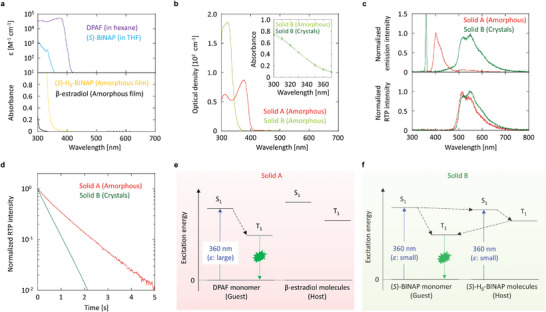
Optical spectral characteristics and RTP generation scheme under excitation. a) Absorption spectra of molecularly dispersed guest (top) and amorphous neat film of the host (bottom). b) Optical density in the amorphous state of solids A and B. Inset represents a change of absorbance measured in an integration sphere between the amorphous state and crystalline state of solid B. c) Steady–state RT emission spectra (top) and afterglow RT emission spectra (bottom) for solids A and solid B. d) Afterglow RT emission decay characteristics of solids A and B. e,f) RTP generation scheme for solid A (e) and solid B (f) under weak excitation at 360 nm.

To investigate the mechanism of irradiance‐induced high‐resolution emission images, the optical properties of solids A and B were measured. Considering the guest chromophores used in solids A and B, DPAF has a large absorption coefficient (*ε*) at 360 nm (**Figure** **2a**, top). (*S*)‐BINAP has a large absorption at wavelengths of less than 373 nm and *ε* at 360 nm is slightly less than 10 M^−1^ cm^−1^ in dispersed conditions. For the host materials used in solids A and B, the amorphous *β*‐estradiol film does not show absorption at 360 nm, whereas the amorphous film of (*S*)‐H_8_‐BINAP has a weak absorption at 360 nm (Figure 2a, bottom). The absorbances of the amorphous film of solid A and the amorphous film of solid B are 0.78 and 0.082 when the thicknesses of the films (*L*) are 10 ± 0.5 µm, respectively (Figure 2b). Therefore, the optical density of the amorphous solid A and amorphous solid B becomes 7.8 × 10^2^ and 0.82 × 10^2^ cm^−1^, respectively (**Table** **1**). Solid B does not show a distinct change in absorbance at 360 nm before and after crystallization from the amorphous state in transmittance measurement, except for the increase of baseline in whole wavelength caused by light scattering of the crystalline film (Figure S5, Supporting Information). The absorbance of the solid B film was measured before and after crystallization from the amorphous state using an integration sphere to exclude the light scattering effect, but no absorbance change was observed (Figure 2b, inset). The amorphous film of solid A shows a fluorescence peak at 401 nm under 360 nm excitation (Figure 2c, top) and generates green afterglow RT emission after ceasing the excitation in ambient conditions (Figure 2c, bottom). The crystalline film of solid B shows a broad emission spectrum with a peak at 549 nm under 360 nm excitation (Figure 2c, top) and generates green afterglow RT emission for a short time after ceasing the excitation (Figure 2c, bottom). The green afterglow RT emission is RTP because of the single exponential decay characteristic (Figure 2d). The average lifetime of RTP (*τ*_p_(RT)) was 1.19 and 0.44 s for the amorphous solid A and the crystalline solid B, respectively. The steady‐state RT emission yield (Φ_e_(RT)) of the amorphous solid A and crystalline solid B were 56% and 13% using 360 nm light with an approximate power of 0.050 mW cm^−2^ for excitation, respectively. Comparing the steady‐state RT emission spectral intensity with the afterglow RTP spectral intensity soon after ceasing the excitation, Φ_p_(RT) values of the amorphous solid A and the crystalline solid B were determined to be 6.2% and 10%, respectively. By subtracting the Φ_p_(RT) value from Φ_e_(RT), the fluorescence yields at RT (Φ_r_^S^(RT)) for the amorphous solid A and crystalline solid B were determined to be 50% and 3.0%, respectively. From the results of absorption spectra (Figure 2a), the excitation light at 360 nm is absorbed by DPAF in solid A (Figure 2e). Because the triplet generation yield (Φ_t_) of DPAF has been measured using transient absorption techniques^[^ ^30^, ^31^, ^32^ ^]^ and can be considered as 1 – Φ_r_^S^(RT) (0.50) because of significant low internal conversion from the lowest singlet excited state (S_1_),^[^ ^33^ ^]^ the lowest triplet excited state (T_1_) of DPAF forms efficiently in amorphous *β*‐estradiol. The T_1_ of DPAF is much less than that of *β*‐estradiol (Figure S6, Supporting Information),^[^ ^4^ ^]^ and the intermolecular triplet quenching is suppressed to generate a green afterglow RTP from DPAF. In the crystalline solid B, the absorption spectra in Figure 2b indicate that excitation at 360 nm was absorbed by the (*S*)‐H_8_‐BINAP host and dispersed by (*S*)‐BINAP (Figure 2f). The S_1_ formed in (*S*)‐BINAP guest molecules not only generates T_1_ in the (*S*)‐BINAP guest but also partially transfers to S_1_ of the (*S*)‐H_8_‐BINAP host. The S_1_ in the (*S*)‐H_8_‐BINAP host leads to the generation of T_1_ in the host and then contributes to the formation of T_1_ in the (*S*)‐BINAP guest. This is because the T_1_ energy of (*S*)‐BINAP is lower than that of (*S*)‐H_8_‐BINAP, as confirmed by optical measurements and quantum chemical calculations (Figures S6 and S7, Supporting Information).^[^ ^29^ ^]^ When the 360 nm light is directly absorbed by (*S*)‐H_8_‐BINAP, the formed S_1_ results in the formation of T_1_ in (*S*)‐BINAP via the intersystem crossing from S_1_ in (*S*)‐H_8_‐BINAP and the T‐T energy transfer from (*S*)‐H_8_‐BINAP to (*S*)‐BINAP. Because the rate constant of phosphorescence (*k*_p_) in (*S*)‐BINAP has been reported as 0.25 s^−1^,^[^ ^29^ ^]^ Φ_t_ of (*S*)‐BINAP in 10 wt.% (*S*)‐BINAP‐doped crystalline (*S*)‐H_8_‐BINAP was determined to be 0.93 by substituting *k*_p_, Φ_p_(RT), and *τ*_p_(RT) into Φ_p_(RT) = Φ_t_ *k*_p_ *τ*_p_(RT). The *k*_p_ of DPAF is also determined to be 0.10 s^−1^ by substituting Φ_p_(RT), Φ_t_, and *τ*_p_(RT) into Φ_p_(RT) = Φ_t_ *k*_p_ *τ*_p_(RT). The *k*_p_ values of DPAF (0.10 s^−1^) and (*S*)‐BINAP (0.25 s^−1^) are comparable to the calculated *k*_p_ values (0.10 and 0.22 s^−1^, respectively). Additionally, the green RTP energy of DPAF and (*S*)‐BINAP could be explained by quantum chemical calculations (Figure S8, Supporting Information). Therefore, we note that the estimated T_1_ generation processes for the guests in Figure 2e and f are reasonable. The total rates of nonradiative deactivation from T_1_ at RT (*k*_nr_) of the amorphous solid A and the crystalline solid B were determined to be 7.4 × 10^−1^ and 2.0 × 10^0^ s^−1^ by substituting *k*_p_ and *τ*_p_(RT) into *τ*_p_(RT) = 1/(*k*_p_ + *k*_nr_), respectively. Because no large differences in Φ_p_(RT), *τ*_p_(RT), Φ_t_, *k*_p_, and *k*_nr_ were observed between the amorphous solid A and crystalline solid B (Table 1), the reason for the significantly suppressed saturation of RTP intensity depending on the excitation intensity remains unclear.

**Table 1:** Photophysical values related to the triplet state.

Sample	Φ_r_^T^(RT)	Φ_t_	Φ_p_(RT)	*τ*_p_(RT)	*Abs*/*L*
				(s)	(×10^2^ cm^−1^)
Film 1	0.12	0.50	0.062	1.19	7.8
Film 2	0.11	0.93	0.10	0.44	0.82

### 2.3 Condensed Accumulation of Triplet Excitons

**Figure 3:**
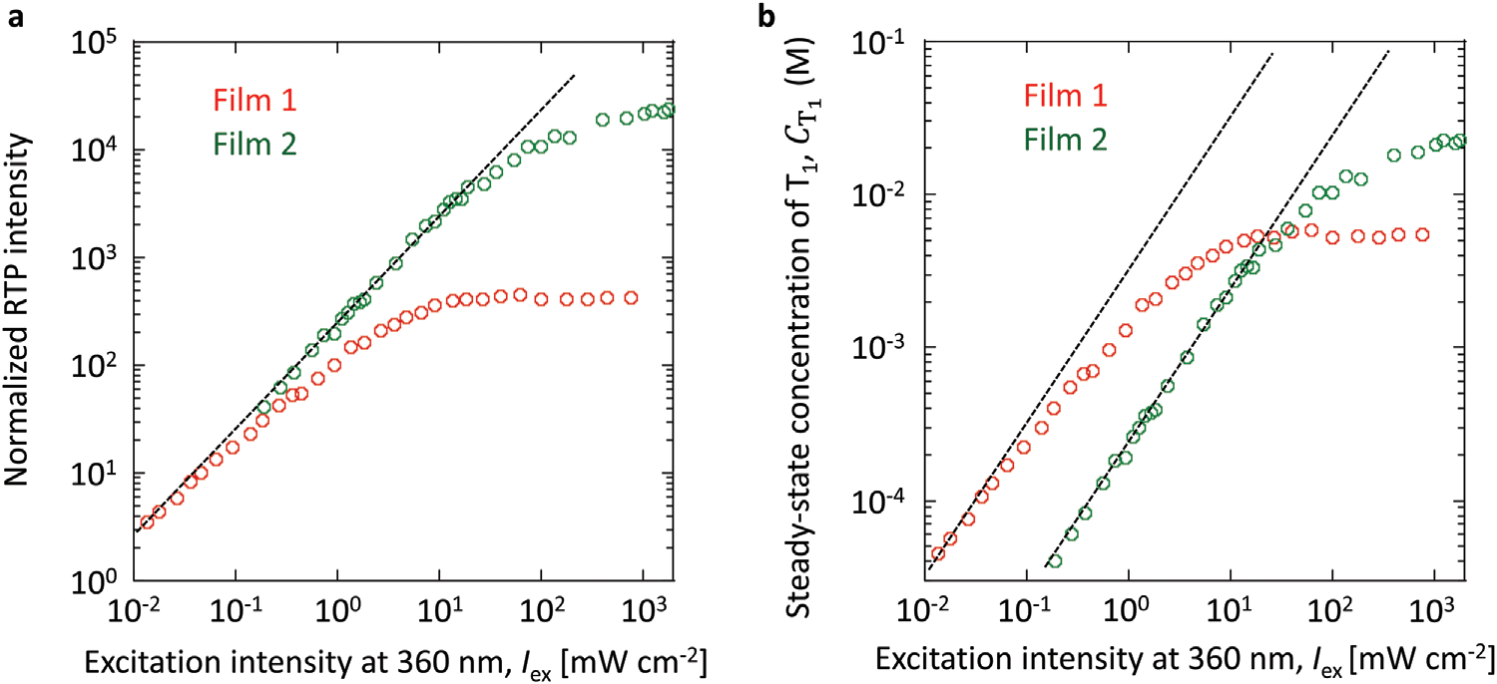
Afterglow RTP intensity and triplet concentration depending on excitation intensity of films 1 and 2. a) The relationship between afterglow RTP intensity and excitation intensity at 360 nm. b) The relationship between continuously accumulated triplet concentration and excitation intensity at 360 nm. Dotted lines represent linear relationship profiles.

The clarification of *ε*, *τ*_p_(RT), and Φ_t_ in Table 1 provides quantitative information on the T_1_ concentration with elevated excitation intensity regarding the amorphous solid A and crystalline solid B, which helps to clarify the different saturation behaviors of RTP intensity between the two solids. To quantitatively discuss the concentration of triplet excitons in solids A and B, a film of amorphous solid A with an absorbance of 0.11 at 360 nm (*Abs*) (film 1) and a film of the crystalline solid B with an absorbance of 0.12 at 360 nm (film 2) were prepared by changing the thicknesses of the films (Figure S9, Supporting Information). The relationship between RTP intensity and excitation intensity was examined for films 1 and 2, using a continuous wave laser at 360 nm as the excitation light. This is because light with wavelengths of less than 350 nm cannot penetrate objective lenses with 100× magnification to increase the light power density and visible light longer than 360 nm does not excite films 1 and 2. The persistent RTP intensity of film 1 is more saturated with elevated irradiance at 360 nm compared with that of film 2 (**Figure** **3a**). The concentration of T_1_ at RT ($C_{T_{1}}$) is expressed in the following equation (see Section S5 in Supporting Information for details):

(1) $$C_{T_{1}}=\frac{E_{p}\left(I_{\mathrm{ex}}\right)}{E_{p}^{{}^{\prime }}\left(I_{\mathrm{ex}}\right)}\;\left[\frac{2.3\times 10^{3}Abs\left(\lambda \right)}{LN_{a}}\right]\Phi_{t}\tau_{p}\left(\mathrm{RT}\right)I_{\mathrm{ex}}$$

where *I*_ex_ is the excitation irradiance at 360 nm, *E*_p_(*I*_ex_) is the RTP intensity at *I*_ex_, and $E_{p}^{\prime }\left(I_{\mathrm{ex}}\right)$ is the RTP intensity at *I*_ex_ if no saturation of RTP occurs with an increase of *I*_ex_ (dotted lines in Figure 3a). By substituting data from Figure 3a for [$\frac{E_{p}\left(I_{\mathrm{ex}}\right)}{E_{p}^{\prime }\left(I_{\mathrm{ex}}\right)}$] and data from Table 1 for $\left[\frac{2.3\times 10^{3}Abs}{LN_{a}}\right]\Phi_{t}\tau_{p}\left(\mathrm{RT}\right)$ into Equation 1, the relationship between $C_{T_{1}}$ and *I*_ex_ is obtained (Figure 3b). We note the relationship between $C_{T_{1}}$ and *I*_ex_ calculated by this technique was comparable to that determined by transient absorption measurements (Section S6 and Figure S10, Supporting Information). As an important point in Figure 3b, we note that $C_{T_{1}}$ of film 1 is saturated at 5.9 × 10^−3^ M, whereas $C_{T_{1}}$ of film 2 increases more with elevated *I*_ex_. Although 1 wt.% (corresponding to approximately 1.8 × 10^−2^ M) DPAF contained in film 1 is the ground state (S_0_), the $C_{T_{1}}$ of DPAF is approximately saturated at 6 × 10^−3^ M with an increase in excitation intensity. When 5 wt.% DPAF is doped into amorphous *β*‐estradiol, the $C_{T_{1}}$ of DPAF does not increase above 6 × 10^−3^ M (Figure S11 and Table S2, Supporting Information). When 10 wt.% DPAF was doped into amorphous *β*‐estradiol, the maximum T_1_ concentration decreased to 2.0 × 10^−3^ M (Figure S11, Supporting Information). This is because annihilation provides an additional quenching pathway at high excitation intensity (Section S8, Figures S12 and S13, Supporting Information). Therefore, the doping concentration is not directly related to maximizing $C_{T_{1}}$. Contrary to the trend in film 1, $C_{T_{1}}$ of film 2 increases up to 2.3 × 10^−2^ M with an increase of *I*_ex_, corresponding to continuous formation of T_1_ than 1 wt.% (1.9 × 10^−2^ M) of (*S*)‐BINAP in the (*S*)‐H_8_‐BINAP crystalline host for film 2. Considering that the previously reported upper limit of the continuous accumulation of $C_{T_{1}}$ under excitation is on the order of 10^−3^ M,^[^ ^27^, ^28^ ^]^ the condensed T_1_ of (*S*)‐BINAP in film 2 is considerably large. Furthermore, the RTP intensity of film 2 is much stronger than that of film 1. Here, we note that films with large absorbance at the excitation wavelength and powder samples cannot be utilized for the estimation of $C_{T_{1}}$ using [$\frac{E_{p}\left(I_{\mathrm{ex}}\right)}{E_{p}^{\prime }\left(I_{\mathrm{ex}}\right)}$], which has been previously reported (Section S9, Figures S14 and S15, Supporting Information).^[^ ^27^, ^28^ ^]^

### 2.4 Exciton Interactions Under Strong Excitation

**Figure 4:**
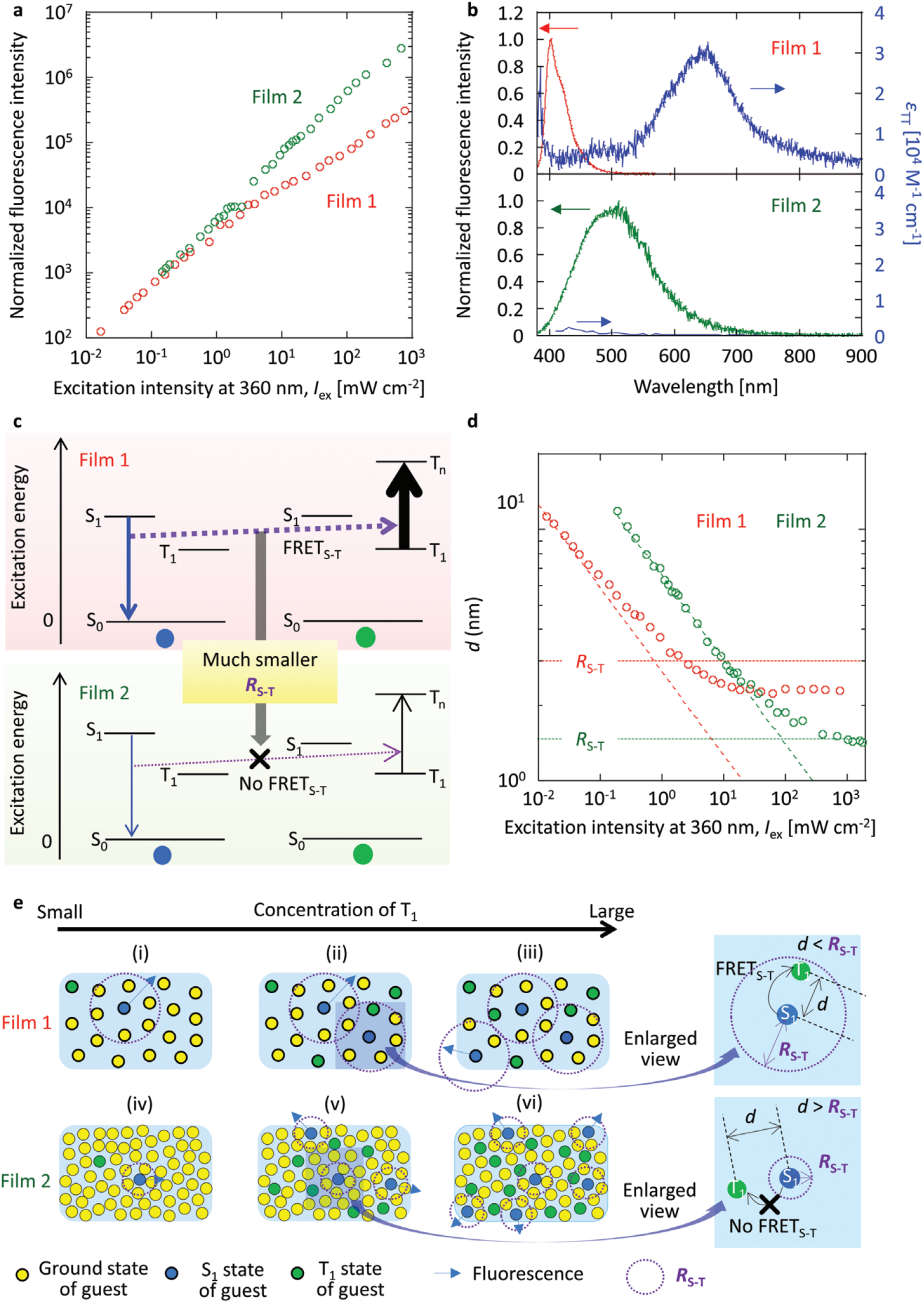
Effect of FRET_S‐T_ on saturation of T_1_ accumulation. a) The relationship between fluorescence intensity and excitation intensity at 360 nm. b) The spectral overlapping between fluorescence and T‐T absorption for 1 wt.% DPAF doped into amorphous β‐estradiol (top) and 10 wt.% (*S*)‐BINAP doped into crystalline (*S*)‐H_8_‐BINAP (bottom). In bottom, *ε*_T‐T_ of (*S*)‐BINAP is data in tetrahydrofuran (THF). c) Jablonski expression to explain a different magnitude of FRET_S‐T_ between film 1 (top) and film 2 (bottom). d) The relationship between *d* and excitation intensity at 360 nm including the *R*_S‐T_(RT) values (dotted line). Dashed lines represent the relationship between *d* and excitation intensity at 360 nm if no saturation of T_1_ occurs with an increase in the excitation intensity. e) Schematic illustrations to explain the different T_1_ accumulation depending on the increase of T_1_ concentration between films 1 and 2 by using *R*_S‐T_(RT).

Although the condensed T_1_ has been observed for film 2, the origin and mechanism of the condensed T_1_ remain to be clarified. The excitation intensity dependence of the fluorescence intensity (*E*_f_) was measured for films 1 and 2. The *E*_f_ of film 1 is saturated by the excitation intensity at 360 nm, whereas the saturation of *E*_f_ is barely observed for film 2 (**Figure** **4a**). To investigate the different fluorescence saturation behaviors, the molar absorption coefficients from T_1_ (*ε*_T‐T_) for DPAF and (*S*)‐BINAP were measured by transient absorption techniques (Figures S16–S18, Supporting Information). The fluorescence spectrum has an overlapping part with the T–T absorption spectrum for dispersed DPAF in amorphous *β*‐estradiol because *ε*_T‐T_ of DPAF is large (Figure 4b, top), and the large *ε*_T‐T_ of DPAF can be checked by quantum chemical calculation (Figure S19, Supporting Information). Therefore, FRET from S_1_ to T_1_ (FRET_S‐T_), is possible when T_1_ accumulates under strong excitation (Figure 4c, top).^[^ ^27^, ^28^ ^]^ Because the FRET_S‐T_ is an additional nonradiative pathway causing a decrease in the Φ_t_ of DPAF, the FRET_S‐T_ becomes a reason to preclude the accumulation of T_1_ in DPAF (Section S12, Supporting Information). However, the spectral overlap between the fluorescence and the T–T absorption is difficult to observe for (*S*)‐BINAP because of the significantly small *ε*_T‐T_ (Figure 4b, bottom), and the significantly small *ε*_T‐T_ across the whole range of visible wavelengths can be verified in the quantum chemical calculation (Figure S19, Supporting Information). Therefore, the weak saturation of *E*_f_ in film 2 indicates that the decrease of Φ_t_ in film 2 caused by the FRET_S‐T_ is small, even under strong excitation irradiance (Figure 4c, bottom). To investigate the decrease of Φ_t_ caused by the FRET_S‐T_, the Förster radius of FRET_S‐T_ (*R*_S‐T_) is considered. Based on the fundamentals of the interaction between the transition dipole moment of fluorescence and the transition dipole moment of T_1_‐T_n_, *R*_S‐T_ (in nm) at RT is given by the following equation:^[^ ^34^, ^35^ ^]^

(2) $$R_{S-T}\left(\mathrm{RT}\right)=0.021{\left[\chi^{2}n^{-4}\Phi_{r}^{S}\left(\mathrm{RT}\right)\right]}^{1/6}$$

where χ^2^ is the orientation factor, *n* ^−1^ is the refractive index, and *J*_S − T_ is the overlapping integral between the fluorescence spectrum of S_1_ and the T–T absorption spectrum of T_1_, which is given by: ^[^ ^34^, ^35^ ^]^

(3) $$J_{S-T}=\frac{\int E_{f}\left(\lambda \right)\varepsilon_{T-T}\left(\lambda \right)\lambda^{4}d\lambda }{\int E_{f}\left(\lambda \right)d\lambda }$$

where *λ* is the wavelength. *R*_S‐T_(RT) of films 1 and 2 are calculated as 3.0 and 1.5 nm, respectively (**Table** **2**). From the plot of $C_{T_{1}}$ versus excitation irradiance (Figure 3b), the average distance between T_1_ (*d*) depending on excitation irradiance was determined for films 1 and 2 (Figure 4d) (Section S13, Supporting Information). When the concentration of S_1_ is much smaller than $C_{T_{1}}$ under steady–state excitation, *d* is considered as the average distance between S_1_ and the accumulated T_1_. In film 1, the decrease of *d* depending on excitation irradiance is saturated at 2.2 nm (Figure 4d, red) because *d* becomes smaller than *R*_S‐T_(RT) under strong excitation. However, *d* can decrease and approach 1.5 nm for film 2 (Figure 4d, green). In films 1 and 2, $C_{T_{1}}$ linearly increases when the value of $C_{T_{1}}$ is small (Figure 4e (i) to (ii) and (iv) to (v), respectively). However, when $C_{T_{1}}$ becomes somewhat large, some of the generated T_1_ is located inside the *R*_S‐T_(RT) of S_1_ for film 1 (Figure 4e (ii)). Therefore, FRET_S‐T_ from S_1_ to the accumulated T_1_ partially occurs as a new S_1_ deactivation pathway and T_1_ is difficult to accumulate for film 1 (Figure 4e (ii) to (iii)). However, more accumulation of T_1_ is possible for film 2 because it is more difficult for T_1_ to fall inside the range of *R*_S‐T_(RT) for S_1_ because of the smaller *R*_S‐T_(RT) (Figure 4e (v) to (vi)). Thus, the accumulated T_1_ does not become a quencher for S_1_ generated by photoexcitation, and less FRET_S‐T_ contributes to the condensed T_1_ of film 2.

**Table 2:** Summary of physical parameters relating to Förster radius of FRET_S‐T_ and FRET_T‐T_. *n* = 1.5 was set as a common value of organic compound to approximate *R*_S‐T_(RT) and *R*_T‐T_(RT). χ^2^ = 0.476 because a randomly distributed transition dipole model is used for the approximation of *R*_S‐T_.^[^ ^36^ ^]^ The change of *R*_S‐T_(RT) and *R*_S‐T_(RT) depending on the change of *n* value from 1.17 to 2.00 is less than ±18% of the values.

Sample	Φ_r_^S^(RT)	Φ_r_^T^(RT)	*ε*_T‐T_	*J*_S‐T_	*J*_T‐T_	*R*_S‐T_(RT)	*R*_T‐T_(RT)
			(M^−1^ cm^−1)^	(nm^4^ M^−1^ cm^−1^)	(nm^4^ M^−1^ cm^−1^)	(nm)	(nm)
Film 1	0.50	0.12	3.1 × 10^4^ ^a)^	2.1 × 10^14^	1.8 × 10^15^	3.0	3.4
Film 2	0.030	0.11	1.3 × 10^3^ ^b)^	4.1 × 10^13^ ^c)^	3.5 × 10^13^ ^c)^	1.5 ^c)^	1.8 ^c)^

^a)^ Value at 650 nm

^b)^ Value at 430 nm

^c)^ Values using *ε*_T‐T_ of (*S*)‐BINAP dissolved in THF.

**Figure 5:**
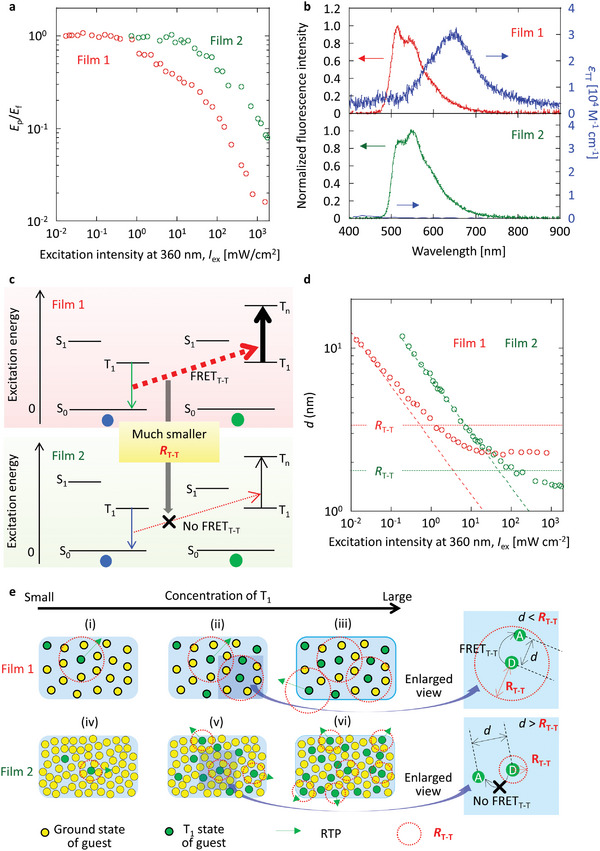
Effect of FRET_T‐T_ on saturation of T_1_ accumulation. a) The relationship between *E*_p_/*E*_f_ and excitation intensity at 360 nm. b) The spectral overlapping between RTP and T‐T absorption for 1 wt.% DPAF doped into amorphous β‐estradiol (top) and 10 wt.% (*S*)‐BINAP doped into crystalline (*S*)‐H_8_‐BINAP (bottom). On the bottom, *ε*_T‐T_ of (*S*)‐BINAP is data in THF. c) Jablonski expression to explain a different magnitude of FRET_T‐T_ between film 1 (top) and film 2 (bottom). d) The relationship between *d* and excitation intensity at 360 nm including the *R*_T‐T_ values (dotted line). Dashed lines represent the relationship between *d* and excitation intensity at 360 nm if no saturation of T_1_ occurs with an increase in the excitation intensity. e) Schematic illustrations to explain the different T_1_ accumulation depending on the increase of T_1_ concentration between films 1 and 2 by using *R*_T‐T_.

A large difference in annihilation among the accumulated T_1_ also causes the large difference in saturation of RT triplet excitons between films 1 and 2. Considering the fluorescence intensity (*E*_f_) and RTP intensity (*E*_p_), the relationship between *E*_p_/*E*_f_ and excitation irradiance was plotted, as shown in **Figure** **5a**, where *E*_p_/*E*_f_ values were normalized to 1 when *E*_f_ and *E*_p_ linearly increase with excitation irradiance. A decrease from 1 for *E*_p_/*E*_f_ versus *I*_ex_ indicates that the generated T_1_ is deactivated via T‐T interactions and/or other photophysical processes. For film 1, *E*_p_/*E*_f_ significantly decreases with the excitation irradiance. Although the decrease was also observed in film 2, the magnitude of the decrease was smaller than that in film 1. To investigate the different triplet depletion behaviors, the spectral overlap between the phosphorescence and T–T absorption was depicted for the DPAF and (*S*)‐BINAP (Figure 5b). The large spectral overlap between RTP and T–T absorption was observed for DPAF in film 1 (Figure 5b, top), whereas no overlapping was observed for (*S*)‐BINAP in film 2 because of the significantly small *ε*_T‐T_ across the whole range of visible wavelengths (Figure 5b, bottom). Therefore, this suggests that the photogenerated T_1_ under strong excitation is largely depleted by the FRET between the T_1_‐S_0_ transition and T_1_‐T_n_ transition (FRET_T‐T_), at least for film 1 (Figure 5c, top). Although the triplet depletion caused by FRET_T‐T_ in film 2 may be suppressed compared with that in film 1 (Figure 5c, bottom), we note that triplet depletion in film 2 is still observed. To quantitatively discuss the triplet depletion caused by FRET_T–T_, the Förster radius of FRET_T‐T_ (*R*_T‐T_) is considered. Based on the fundamental interaction of the transition dipole moment of radiation from T_1_ and the transition dipole moment of T_1_‐T_n_, *R*_T‐T_ (in nm) at RT is considered using the following equation:^[^ ^37^ ^]^

(4) $$R_{T-T}\left(\mathrm{RT}\right)=0.021{\left[\chi^{2}n^{-4}\Phi_{r}^{T}\right]}^{1/6}$$

where Φ_r_^T^(RT) is the radiation yield from T_1_ at RT in the absence of FRET_T‐T_, and *J*_T − T_ is the overlapping integral between the phosphorescence spectrum from T_1_ and the T–T absorption spectrum of T_1_. Φ_r_^T^(RT) is defined as Φ_r_^T^(RT) = Φ_p_(RT)/Φ_t_.^[^ ^37^ ^]^ *J*_T − T_ is given by:

(5) $$J_{T-T}\;\left(\lambda \right)=\frac{\int E_{p}\left(\lambda \right)\varepsilon_{T-T}\left(\lambda \right)\lambda^{4}d\lambda }{\int E_{p}\left(\lambda \right)d\lambda }$$

The *R*_T–T_(RT) values of films 1 and 2 were determined to be 3.4 and 1.8 nm, respectively (Table 2). In film 1, a decrease of *d* becomes saturated with excitation intensity when *d* is less than *R*_T–T_(RT) (Figure 5d, red). For film 2, *d* can become smaller when the excitation intensity is strong, at least for a sufficiently small *R*_T–T_(RT) (Figure 5d, green). When $C_{T_{1}}$ is less than 10^−4^ M, $C_{T_{1}}$ linearly increases with excitation intensity for both films 1 and 2 (Figure 5e (i) and (ii) and (iv) and (v), respectively). However, one T_1_ of DPAF starts to fall inside the *R*_T–T_ of another T_1_ of DPAF for film 1 when $C_{T_{1}}$ increases more than 10^−3^ M (Figure 5e (ii)). Therefore, photogenerated T_1_ is partially deactivated by FRET_T–T_ between two T_1_, and the saturation of $C_{T_{1}}$ occurs (Figure 5e (ii) and (iii)). However, because of the much smaller *R*_T‐T_(RT) for (*S*)‐BINAP in film 2, $C_{T_{1}}$ increases and allows for the small *d* corresponding to condensed T_1_ (Figure 5e (v) and (vi)). Therefore, the small *ε*_T–T_ of (*S*)‐BINAP minimizes the triplet depletion of (*S*)‐BINAP under strong excitation. Notably, the spectral overlap between fluorescence and T–T absorption (Figure 4b, top) and the spectral overlap of RTP and T‐T absorption (Figure 5b, top) are not large for DPAF. Even in this situation, the triplet exciton accumulation with increasing excitation irradiance stops at the magnitude of 10^−3^ M. Therefore, we note the continuous triplet accumulation on the order of 10^−2^ M is significant. Although the small *ε*_T–T_ of (*S*)‐BINAP minimizes the triplet depletion of (*S*)‐BINAP under strong excitation, triplet depletion of film 2 is still observed (Figure 5a). In recent years, triplet depletion via photoinduced ionization has received significant attention.^[^ ^38^, ^39^, ^40^, ^41^ ^]^ Although the continuous T_1_ accumulation over 2 × 10^−2^ M can be observed for 10 wt.% (*S*)‐BINAP‐doped (*S*)‐H_8_‐BINAP crystals (film 2), our quantitative analysis indicates that photoinduced ionization becomes an additional triplet depletion pathway for the material under strong excitation (Section S14 and Figure S20, Supporting Information). Therefore, the suppression of photoinduced ionization, as well as the FRET_S‐T_ and FRET_T‐T_, is crucial to consider for developing continuous triplet accumulation over 10^−1^ M.

### 2.5 Persistent RTP from Individual Particles in Aqueous Media

When materials smaller than the diffraction limit are used, the upper limit of the brightness of persistent RTP is proportional to *k*_p_ $C_{T_{1}}$ *V* (Section S15, Supporting Information), where *V* is the volume of material. When two materials with the same *V* are compared, a material with larger *k*_p_ $C_{T_{1}}$ shows brighter afterglow RTP. The maximum of $C_{T_{1}}$is 5.9 ×10^−3^ and 2.3 ×10^−2^ M for solids A and B, respectively. Therefore, the maximum value of *k*_p_ $C_{T_{1}}$ of solid B is approximately 10 times larger than that of solid A because *k*_p_ of solids A and B is 0.10 and 0.25 s^−1^, respectively. Consequently, the afterglow RTP brightness of solid B is much larger than that of solid A in microscope images under strong excitation irradiance, which enables the irradiance‐induced appearance of the afterglow image in Figure 1c. Although the brightness of afterglow RTP from individual particles can be increased using a larger *k*_p_, the afterglow characteristics disappear if *k*_p_ is too large. Therefore, the increase of $C_{T_{1}}$ is a crucial factor for producing high‐resolution afterglow characteristics.

**Figure 6:**
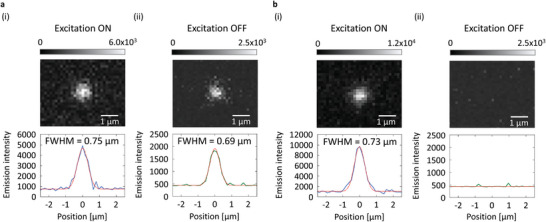
Microscope emission images (top) and resolution profiles (bottom) of individual particles under excitation (i) and after ceasing the excitation (ii). a) Individual particle of 10 wt.% (*S*)‐BINAP‐doped (*S*)‐H_8_‐BINAP crystals. b) Individual particle of 1 wt.% DPAF‐doped (*S*)‐H_8_‐BINAP crystals. Excitation intensity and wavelength are 350 mW cm^−2^ and 360 nm, respectively. In the bottom graphs, red dotted lines represent fitting lines based on Gaussian distributions.

In addition, the nanostructures of materials showing RTP have been checked using transmission electron microscopy. However, afterglow images have generally been demonstrated for bulk aggregates of the nanomaterials with sizes of 10 µm or more because the afterglow brightness of individual nanomaterials in aqueous conditions can be weak.^[^ ^13^, ^17^ ^]^ For solid B, afterglow RTP from an individual particle with a size approaching the diffraction limit of green light is observed because of the large upper limit of $C_{T_{1}}$ (**Figure** **6** **a**). The full‐with‐half maximum (FWHM) of persistent RTP from individual particles of solid B is 0.69 µm, which is determined using Gaussian fitting of data in Figure 6a (ii). Because the diffraction limit of green emission at 549 nm under objective lens (NA = 1.3) is 0.29 µm based on 549/2NA, the resolution of the persistent RTP of solid B approaches the diffraction limit. Because solid B maintains a crystalline state (Figure S21a, Supporting Information) and does not dissolve in water, the afterglow RTP is observed for a long time in aqueous conditions. We note that the afterglow emission from individual particles with sizes approaching the diffraction limit in aqueous conditions has not been previously reported. When 1 wt.% DPAF is doped into crystalline (*S*)‐H_8_‐BINAP, fluorescence from the individual crystalline particles with sizes approaching the diffraction limit of green light (Figure S21b, Supporting Information) was observed in high‐resolution emission imaging under excitation at 360 nm (Figure 6b (i)) while persistent RTP from individual particles could not be detected after the 360 nm excitation ceased (Figure 6b (ii)). The afterglow image from individual nanoparticles in this paper is an important step for high‐resolution imaging independent of autofluorescence using cost‐effective and small‐scale photodetectors in ambient aqueous conditions.

## 3 Conclusion

Autofluorescence‐free luminescence imaging from individual nano‐sized objects has yet to be achieved. This is because the afterglow brightness of typical long‐persistent emitters exhibits a delayed emission time of ≈1 h, increasing with increasing excitation light intensity.^[^ ^26^ ^]^ *p*RTP can potentially produce stronger afterglow emission under intense excitation.^[^ ^26^ ^]^ Although afterglow detection of individual nano‐sized materials requires further suppression of saturation of *p*RTP at high excitation light intensity, no key factors or materials that mitigate this suppression have been reported. In this paper, it is clarified that excitation FRET_S–T_ and FRET_T–T_ are dominant processes that hinder bright afterglow, based on analysis of the Förster radius of FRET_S–T_ and FRET_T–T_. Furthermore, to minimize FRET_S‐T_ and FRET_T–T_, 10 wt.% (*S*)‐BINAP‐doped (*S*)‐H_8_‐BINAP crystalline materials with minimized T‐T absorption coefficients were found. The suppression of both FRET_S–T_ and FRET_T–T_, owing to (*S*)‐BINAP having a small *ε*_T–T_, leads to a continuous accumulation concentration of triplet states above 1 wt.% (2.3 × 10^−2^ M) for 10 wt.% (*S*)‐BINAP‐doped (*S*)‐H_8_‐BINAP crystalline materials. The concentration of the long‐lived triplet excitons measured in this paper is an order of magnitude greater than previously reported. Even if Φ_p_ is large, if *p*RTP intensity saturates as soon as the excitation light intensity increases, the materials cannot be used for high‐resolution imaging. Therefore, this paper clarifies the need to quantify the Förster radius of FRET_S–T_ and FRET_T–T_ as well as Φ_p_. Because of the suppression of dual FRET_S–T_ and FRET_T–T_ owing to the (*S*)‐BINAP with small *ε*_T–T_, the concentration of triplet state continuously accumulates above 1 wt.% for 10 wt.% (*S*)‐BINAP‐doped (*S*)‐H_8_‐BINAP crystalline materials. Because of the condensed T_1_ of (*S*)‐BINAP, bright green afterglow RTP was observed, which contributed to the observation of afterglow RTP in aqueous conditions from individual particles of 10 wt.% (*S*)‐BINAP‐doped (*S*)‐H_8_‐BINAP crystalline material. In addition, condensed T_1_ of (*S*)‐BINAP allowed the irradiance‐dependent appearance of high‐resolution afterglow images. This work demonstrates a novel type of anticounterfeiting. Furthermore, analysis of the maximum triplet concentration is crucial to identify the origin of the phosphorescent luminogens in conjugated molecular crystals. Recently, several reports have determined that the persistent RTP of conjugated molecular crystals is caused by impurities contained in the conjugated molecular crystals.^[^ ^42^, ^43^, ^44^, ^45^ ^]^ The concentration of the triplet state of the dopant chromophore does not exceed the concentration of the ground state of the chromophore. Thus, the concentration of the triplet state does not largely increase when the impurity with commonly low concentration is RTP luminogen (Figure S22, Supporting Information).

## Conflict of Interest

The authors declare no conflict of interest.

## Supporting information

Supporting Information

Supplemental Movie 1

Supplemental Movie 2

## Data Availability

The data that support the findings of this study are available in the supplementary material of this article.
