# Supplementary material for: Continuous Condensed Triplet Accumulation for Irradiance‐Induced Anticounterfeit Afterglow
Source: Adv Sci (Weinh). 2023 Oct 28;10(36):2304374. doi: 10.1002/advs.202304374 (PMC10754144; doi:10.1002/advs.202304374)
Supplement: Supplementary file 1 — Supporting Information [file ADVS-10-2304374-s002.pdf]

ADVANCED SCIENCE
Open Access

Supporting Information

for *Adv. Sci.*, DOI 10.1002/advs.202304374

Continuous Condensed Triplet Accumulation for Irradiance-Induced Anticounterfeit Afterglow

*Ende Hopsah Badriyah, Kikuya Hayashi, Bahadur Sk, Rina Takano, Takayuki Ishida and Shuzo Hirata**

Supporting Information

Continuous condensed triplet accumulation for irradiance-induced anticounterfeit afterglow

Ende Hopsah Badriyah, Kikuya Hayashi, Bahadur Sk,

*Rina Takano, Takayuki Ishida, Shuzo Hirata**

*Corresponding author. E-mail: shuzohirata@uec.ac.jp

Contents

S1. Experimental section

S2. Dispersion condition of (*S*)-BINAP in (*S*)-H_8-BINAP crystalline lattice

S3. Additional demonstration

S4. Optical properties

S5. Derivation of Equation 1

S6. Estimation of triplet concentration using transient absorption

S7. Consideration of maximum T_1 concentration at different dopant concentrations

S8. Suppression of T_1 accumulation by triplet-triplet annihilation owing to triplet migration at high guest concentration

S9. Mandatory points to be checked to determine C_{T_1} using the excitation intensity dependence of RTP

S10. Measurement procedure of ε_{T-T}

S11. Quantum chemical calculation of ε_{T-T}

S12. Potential mechanism by which $FRET_{T-S}$ lowers triplet generation yield

S13. Calculation of *d*

S14. Effect of photoinduced ionization under strong excitation irradiance

S15. Theoretical RTP brightness of individual particles with sizes below the diffraction limit

S16. Transmission electron microscope images of individual crystals

S17. Maximum triplet concentration when the dopant concentration is small

S18. Supporting movies

S19. Supporting references

S1. Experimental section.

Preparation of samples

9,9-Dimethyl-N^2,N^7-diphenyl-N^2,N^7-di-*m*-tolyl-9*H*-fluorene-2,7-diamine (DPAF) (Tokyokasei Chemical Industries (TCI), Tokyo, Japan) and β-estradiol (TCI) were mixed in the ratio of x wt% and 100 − x wt%, respectively. The powder was melted and then quenched to room temperature (RT) to prepare amorphous solid A. (*S*)-(–)-2,2'-Bis(diphenylphosphino)-5,5',6,6',7,7'8,8'-octahydro-1,1'-binaphthyl [(*S*)-H_8-BINAP] (Sigma Aldrich, St. Louis, Missouri, USA) was purified by column chromatography. (*S*)-(-)-2,2'-Bis(diphenylphosphino)-1,1'-binaphthyl [(*S*)-BINAP] (Sigma Aldrich) powder and the purified (*S*)-H_8-BINAP powder were mixed in the ratio of 10 and 90 wt%, respectively. The powder was melted and then quenched to RT to obtain a 10 wt% (*S*)-BINAP-doped (*S*)-H_8-BINAP amorphous solid. The amorphous solid was placed in a dichloromethane (DCM) vapor atmosphere for 3 min to obtain a polycrystalline solid B.

For the samples of the demonstration in Figure 1, polycrystalline solid B was spread out by mechanical grinding on a microscope cover glass substrate (Figure 1b (i)). The remaining procedures to complete the anticounterfeit media are explained in the main text. For the sample of the demonstration in Figure S4, small amounts of crystalline solid B were dispersed on the powder film of solid A on a glass substrate.

For samples to determine the molar absorption coefficient from the ground state by transmittance measurements, solid A or solid B was injected between two microscope cover glass substrates with a gap of 10 µm and set on a hot plate at 240 °C. The samples are quenched to RT to obtain two amorphous films of solid A and solid B with thicknesses (*L*) of 10 µm. The two amorphous films were used to determine the optical densities at 360 nm (*Abs*/*L*), as shown in Figure 2b.

Regarding the measurement of the emission quantum yield and the emission characteristics shown in Figure 2c and 2d, solid A or solid B were placed on a quartz substrate on a hot plate at 240 °C. After solid A or solid B melted, another quartz substrate was placed onto the materials to spread out the melted materials between the two quartz substrates. The sandwiched samples were quenched to RT to obtain an amorphous state of solid A or solid B. The amorphous solid A between two quartz substrates was used for the emission quantum yield and emission characteristics of 1 wt% DFAP-doped amorphous β-estradiol in Table 1, Table 2, Figure 2c, and Figure 2d. For the amorphous solid B between two quartz substrates, one quartz substrate was peeled off and then the quartz substrate coated with an amorphous layer of solid B was placed in a DCM vapor atmosphere to crystallize solid B. The crystalline solid B on a quartz substrate was used

to obtain the emission quantum yield and emission characteristics of 10 wt% (*S*)-BINAP-doped crystalline (*S*)-H_8-BINAP in Table 1, Table 2, Figure 2c, and Figure 2d.

Regarding the preparation of films 1 and 2 to obtain the excitation intensity dependence of emission intensity in Figure 3a and 4a, solid A was injected between two microscope cover glass substrates on a hot plate at 240 °C and then quenched to RT to obtain film 1 (1 wt% DPAF-doped amorphous *β*-estradiol layer between two thin glass substrates). For film 2, solid B was injected between two microscope cover glass substrates on a hot plate at 240 °C and then quenched to RT to obtain the amorphous layer of 10 wt% (*S*)-BINAP-doped (*S*)-H_8-BINAP between two thin glass substrates. The sandwiched sample was placed in DCM vapor for a long time to crystallize 10 wt% (*S*)-BINAP-doped (*S*)-H_8-BINAP to prepare film 2 (10 wt% (*S*)-BINAP-doped crystalline (*S*)-H_8-BINAP layer between two thin glass substrates). The absorbances at 360 nm caused by photon absorption of films 1 and 2 were 0.106 and 0.122, respectively (Figure S9).

Individual particles for transmission electron microscope (TEM) images were prepared using a previously reported top-down process[S1] as follows. Polycrystals of solid B were dispersed in an aqueous solution of 3.3 mg-poly(ethylene glycol)-*block*-poly(propylene glycol)-*block*-poly(ethylene glycol) diacrylate (Sigma Aldrich) dissolved in 1 ml of water. After the solution was dispersed using a sonicator (UR-20P, Tomy Seiko Co., LTD, Tokyo, Japan), the solution was filtrated using a membrane filter (Millex®-SV, PVDF, 5.0 µm, Merck, Darmstadt, Germany) to obtain small particles of 10 wt% (*S*)-BINAP-doped (*S*)-H_8-BINAP dispersed in water. Next, DPAF powder and the purified (*S*)-H_8-BINAP powder were mixed in the ratio of 1 and 99 wt%, respectively. The powder was melted and then quenched using liquid nitrogen. Scratching the solid at RT produced polycrystalline solids of 1 wt% DPAF-doped (*S*)-H_8-BINAP. The polycrystals of 1 wt% DPAF-doped (*S*)-H_8-BINAP were dispersed in an aqueous solution of 3.3 mg-poly(ethylene glycol)-*block*-poly(propylene glycol)-*block*-poly(ethylene glycol) diacrylate (Sigma Aldrich) dissolved in 1 ml of water. After the solution was dispersed using a sonicator (UR-20P, Tomy Seiko Co., LTD), the solution was filtrated using a membrane filter (Millex®-SV, PVDF, 5.0 µm, Merck) to obtain small particles of 1 wt% DPAF-doped (*S*)-H_8-BINAP dispersed in water.

Single crystalline analysis

(*S*)-H_8-BINAP (Sigma Aldrich) was purified by column chromatography. The purified (*S*)-H_8-BINAP powder and (*S*)-BINAP (Sigma Aldrich) powder were mixed in the ratio of 90 and 10 wt%, respectively. The mixed powder was sublimated using 10^{-4} torr at 230 °C to form single crystals of (*S*)-BINAP doped into the (*S*)-H_8-BINAP crystalline

lattice. Although single crystals of the purified (*S*)-H_8-BINAP did not show persistent RT emission at 360 nm excitation, the crystals prepared using the mixed powder of (*S*)-BINAP and (*S*)-H_8-BINAP presented a strong green afterglow RT emission. X-Ray diffraction data of (*S*)-H_8-BINAP single crystal and 10 wt% (*S*)-BINAP-doped (*S*)-H_8-BINAP single crystal were collected on a CCD diffractometer (Saturn70, Rigaku, Tokyo, Japan) with graphite monochromated Mo Kα radiation (λ = 0.71073 Å). Suitable crystals of 0.20 mm × 0.17 mm × 0.13 mm for (*S*)-H_8-BINAP and 0.67 mm × 0.45 mm × 0.10 mm for 10 wt% (*S*)-BINAP-doped in (*S*)-H_8-BINAP were selected and mounted on a suitable support using an XtaLAB Synergy R, DW system, HyPix diffractometer (V1.171.42.72a, 2022, Rigaku). The crystal was kept at a steady temperature of 26 °C during data collection. The structure was solved using the ShelXT 2018/2 (Sheldrick, 2018) structure solution program with the Intrinsic Phasing solution method and Olex2 as the graphical interface.[S2] The model was refined using version 2018/3 of ShelXL 2018/3 (Sheldrick, 2015) with least squares minimization. CCDC reference numbers 2263276 and 2263371 include the experimental details and full geometrical parameter tables for the two crystals. Comparable lattice information of (*S*)-H_8-BINAP was observed for the (*S*)-BINAP-doped (*S*)-H_8-BINAP crystal, as well as the pristine (*S*)-H_8-BINAP crystal (Figure S1 and Table S1). However, the distinct variation of the cell volume of (*S*)-H_8-BINAP crystal by doping (*S*)-BINAP (Table S1) suggests the random doping of (*S*)-BINAP in (*S*)-H_8-BINAP host due to similar molecular structure. Indeed, thin chromatography using one single crystal of the (*S*)-BINAP-doped (*S*)-H_8-BINAP crystals indicates the existence of both (*S*)-BINAP and (*S*)-H_8-BINAP (Figure S1). Nuclear magnetic resonance (NMR) spectra of the (*S*)-BINAP-doped (*S*)-H_8-BINAP crystals indicate that the 10 wt% (S)-BINAP exists in the (*S*)-BINAP-doped (*S*)-H_8-BINAP crystals (Figure S2). The overall results in Figure S1 and S2 are explained by (*S*)-H_8-BINAP molecules in the crystalline lattice of (*S*)-H_8-BINAP being randomly replaced by (*S*)-BINAP molecules. Because the size and shape of (*S*)-BINAP are comparable to that of (*S*)-H_8-BINAP, efficient replacement of (*S*)-H_8-BINAP by (*S*)-BINAP is possible. The molecular doping in other molecular crystalline lattices has been reported for fluorescence lasing materials[S3] and persistent phosphorescence materials[S4]. We note that the RTP spectral shape, the RTP yield, and the RTP lifetime of the thin films and single crystals are comparable (Figure S3).

Optical measurements

Absorption spectra of films 1 and 2 were collected using an absorption spectrometer (V-760, Jasco, Ltd., Tokyo, Japan). The absorbances of the amorphous powder and crystalline powder of solid B were measured using an absolute luminescence quantum

yield measurement system (G9920-02G, Hamamatsu Photonics, Shizuoka, Japan). The emission yield of the steady-state excitation was determined using the absolute PL measurement system (G9920-02G, Hamamatsu Photonics). The ratio of the fluorescence and afterglow RTP was determined using a photonic multichannel analyzer (C10027-01, Hamamatsu Photonics) as a photodetector and light from an excitation unit of a fluorometer (P-8300, Jasco) to determine the fluorescence (Φ_r^S) and phosphorescence (Φ_P) yields at RT. The emission lifetimes up to 40 ms were measured using a multichannel analyzer (C10027-01, Hamamatsu Photonics) as a photodetector and light from an excitation unit of a fluorometer (FP-8300, Jasco). The molecular absorption coefficient of triplet-triplet (T-T) absorption ($\varepsilon_{T\text{-}T}$) was measured using a sub-nanosecond transient absorption spectrophotometer (picoTAS, Unisok, Osaka, Japan) by second harmonic generation from a 532 nm Q-switched microchip laser (PNV-M02510-1×0, Teem Photonics, Meylan, France) and a 355 nm Q-switched microchip laser (PNV-M02510-1×0, Teem Photonics, Meylan, France). To measure T-T absorption of the film sample, a continuous-wave laser emitting at 360 nm (UV-F-360, CNI, Changchun, China) and UV-VIS-NIR light source (DT-MINI-2-GS, Ocean Optics, Tokyo, Japan) were used as the excitation and white probe light, respectively. A photonic multichannel analyzer (C10027-01, Hamamatsu Photonics) was used as a photodetector for the T-T absorption measurement. Optical setup of the transient absorption under contineous excitation is shown in Figure S10a.

Microscopic optical measurements

The relationship between emission intensity and excitation intensity was measured using an inverted optical microscope (IX73, Olympus, Tokyo, Japan) with an oil immersion objective lens (UPlan FLN×100/1.3 NA, oil, Olympus). A continuous-wave laser emitting at 360 nm (UV-F-360, CNI) was used as an excitation source to obtain epi-emission signals. The excitation beam power was measured using a photodiode power sensor (S130VC, Thorlabs, New Jersey, USA). The excitation area used to determine the excitation power density was determined by observing the emission area from films 1 and 2. A dichroic mirror (FF470-Di01, Semrock, New York, USA) and a long-pass filter (LP02-473RU, Semrock) were used to collect emission signals from films 1 and 2. Schematic illustration of optical setup is shown in Figure 1d. Steady-state RT emission intensity depending on excitation intensity under the 360 nm excitation was measured. Persistent RTP intensity depending on excitation intensity was determined from emission intensity within 40 ms after completely ceasing the 360 nm excitation for Figure 3a. By substructing the persistent emission intensity from the steady-state RT emission intensity, the excitation intensity dependence of the fluorescence intensity was determined for

Figure 4a. An objective lens (UPlanAPO ×10/0.40 NA, Olympus) was used to obtain the images in the demonstration of Figure 1d.

Electron microscope

Images of individual nanoparticles of 1 wt% DPAF-doped (*S*)-H_8-BINAP and 10 wt% (*S*)-BINAP-doped (*S*)-H_8-BINAP were obtained using an Environmental TEM (H-9500, Hitachi High-Tech Corporation, Tokyo, Japan).

Quantum chemical calculation

Optimized T_1 geometry was calculated using density functional theory (DFT) (Gaussian09/B3LYP/6-31G(d)). From the geometry, T-T absorption characteristics were calculated using time-dependent DFT (Gaussian09/B3LYP/6-31G(d)). Single-point calculations were performed using the Amsterdam Density Functional (ADF) 2018 software package for the optimized T_1 geometries to determine the T_1-S_0 energy of the chromophores. For the calculation of k_p in DPAF and (*S*)-BINAP, the spin-orbit matrix elements were treated as a perturbation based on scalar relativistic orbitals using the PBE0 functional and TZP basis sets. The scalar relativistic-time-dependent DFT calculations included 10 singlet + 10 triplet excitations, which were used as the basis for the perturbative expansions in the calculations.

S2. Dispersion condition of (*S*)-BINAP in (*S*)-H8-BINAP crystalline lattice

Table S1. Crystallographic data table for (*S*)-H_8-BINAP and 10 wt% (*S*)-BINAP doped in (*S*)-H_8-BINAP.

Sample	(*S*)-H_8-BINAP	10 wt% (*S*)-BINAP doped in (*S*)-H_8-BINAP
Chemical formula	$C_{44}H_{40}P_2$	$C_{44}H_{40}P_2$
Formula weight	630.70	630.70
Temperature/K	299.1(6)	299.0(3)
Crystal system	orthorhombic	orthorhombic
Space group	$P2_12_12_1$	$P2_12_12_1$
a/Å	9.8493(2)	9.8410(4)
b/Å	17.8904(4)	17.8829(8)
c/Å	19.8598(5)	19.8325(7)
α/°	90	90
β/°	90	90
γ/°	90	90
V/Å^3	3499.45(14)	3490.2(2)
Z	4	4
d/g cm^{-3}	1.197	1.200
μ/mm^{-1}	0.154	0.155
F(000)	1336	1336
Radiation	Mo Kα (λ = 0.71073 Å)	Mo Kα (λ = 0.71073 Å)

2Θ range for data collection/°	4.102 to 63.962	4.724 to 63.758
Index ranges	$-13 \le h \le 12$, $-25 \le k \le 24$, $-29 \le l \le 25$	$-13 \le h \le 14$, $-25 \le k \le 26$, $-28 \le l \le 26$
Reflections collected	50362	53896
Independent reflections	10285 [R_{int} = 0.0328, R_{sigma} = 0.0311]	10099 [R_{int} = 0.0545, R_{sigma} = 0.0413]
Data/restraints/parameters	10285/0/415	10099/0/415
Goodness-of-fit on F^2	0.952	0.985
Final R indexes [$I \geq 2\sigma (I)$]	R_1 = 0.0404, wR_2 = 0.1221	R_1 = 0.0497, wR_2 = 0.1386
Final R indexes [all data]	R_1 = 0.0623, wR_2 = 0.1344	R_1 = 0.0792, wR_2 = 0.1511
Flack parameter	-0.05(2)	0.02(3)
Largest diff. peak/hole / e Å^{-3}	0.29/−0.21	0.29/−0.19
CCDC number	2263276	2263371

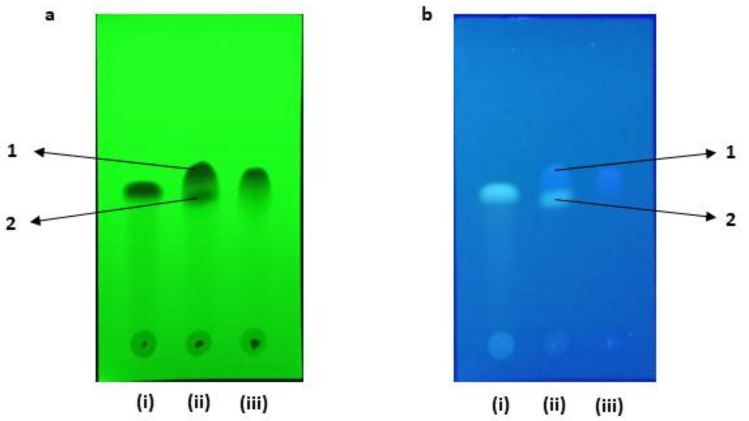

Figure S1. View under (a) 254 nm excitation and (b) 365 nm excitation of three materials (i)-(iii) in thin layer chromatography plates (silica gel) using eluent: hexane/dichloromethane = 6:4 vol. (i) Commercially available (*S*)-BINAP. (ii) Co-crystal of 10 wt% (*S*)-BINAP doped in (*S*)-H_8-BINAP obtained from sublimation. Spots 1 and 2 indicate that (*S*)-BINAP is well dispersed in (*S*)-H_8-BINAP. (iii) (*S*)-H_8-BINAP obtained from purification by column chromatography from the commercially available (*S*)-H_8-BINAP with a purity of more than 94% (Sigma Aldrich).

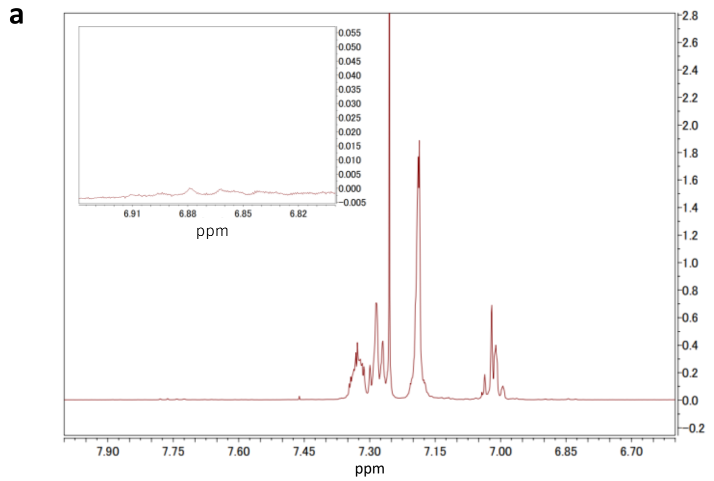

b

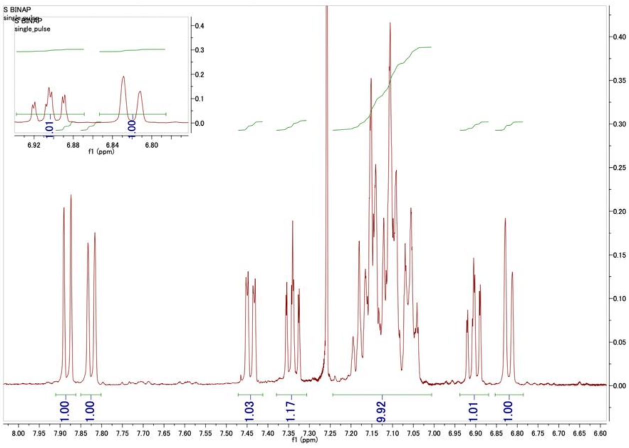

c

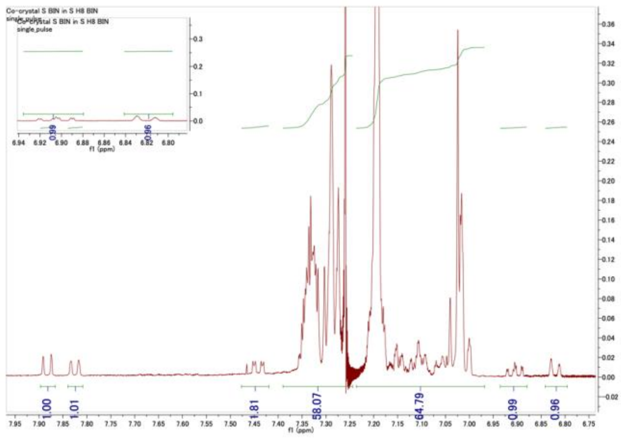

Figure S2. (a) ^{1}H NMR spectrum of (*S*)-H_8-BINAP after purification. (b) ^{1}H NMR spectrum of (*S*)-BINAP. (c) ^{1}H NMR spectrum of co-crystal from the sublimation of 10 wt% (*S*)-BINAP doped in (*S*)-H_8-BINAP.

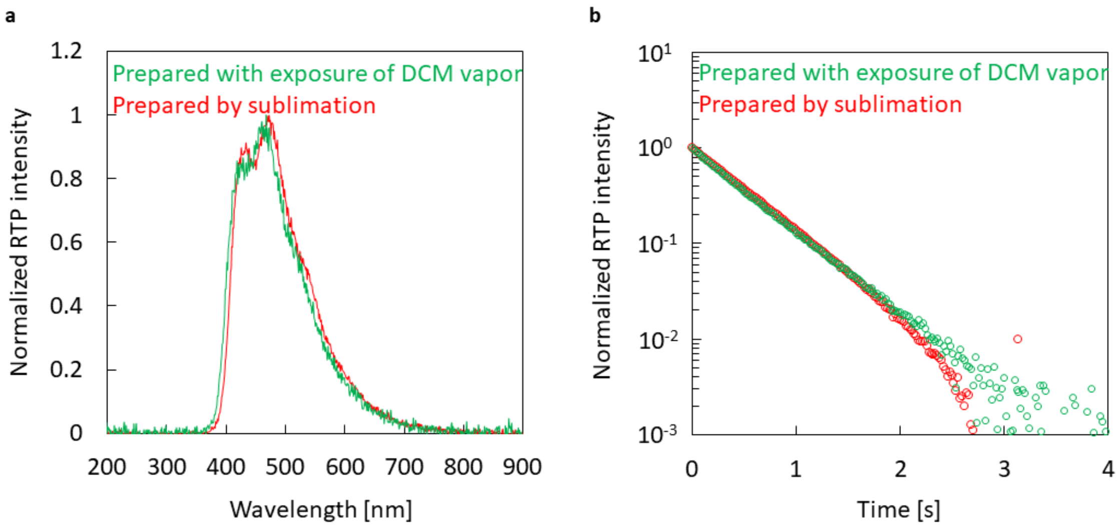

Figure S3. (a) RTP spectra and (b) RTP decay characteristics of two crystals. One is a 10 wt% (*S*)-BINAP-doped (*S*)-H_8-BINAP crystalline film exposed to dichloromethane vapor from an amorphous state. The other is the 10 wt% (*S*)-BINAP-doped (*S*)-H_8-BINAP single crystal, which is prepared by sublimation of mixed powder composed of 10 wt% (*S*)-BINAP and 90 wt% (*S*)-H_8-BINAP.

S3. Additional demonstration

Some grains of solid B (crystals) are placed over solid A (amorphous) powder on a glass substrate and set under a fluorescence microscope (Figure S4a). Although afterglow RTP of the film containing solid A and solid B has been measured soon after ceasing excitation at 360 nm and 0.3 mW cm^{-2}, the location of solids B and A could not be distinguished well (Figure S4b (i)) because they generate comparable intensities of green afterglow emission. However, the afterglow RTP intensity of solid B increases while that of solid A remains small when the excitation intensity at 360 nm increases from 0.3 to 30 mW cm^{-2}. Therefore, the location of solid B with the size of a few microns could be distinguished (Figure S4b (ii)). When the excitation intensity at 360 nm increases to 300 mW cm^{-2}, the afterglow RTP intensity of solid B increases further while the RTP intensity of solid A hardly increases. Therefore, more contrast could be observed to distinguish solid B from solid A (Figure S4b (iii)).

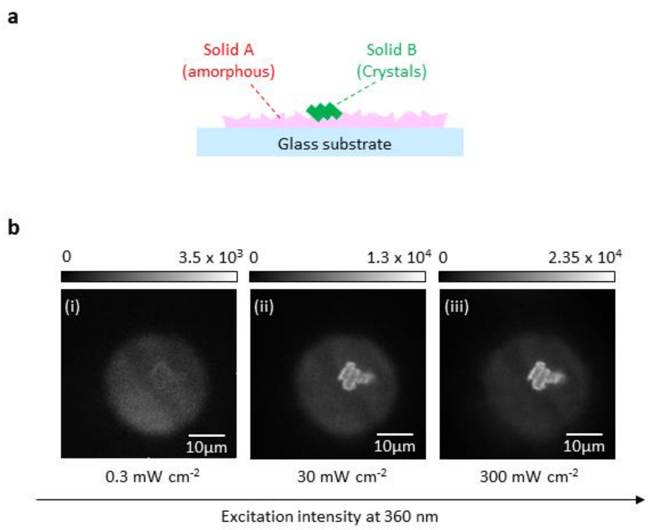

Figure S4. (a) Illustrations of a sample containing solid A and solid B on a glass substrate. (b) The irradiance-dependent appearance of high-resolution afterglow images of the sample. The images are taken in air by counting emission signals generated within 50 ms after ceasing the 360 nm excitation.

S4. Optical properties

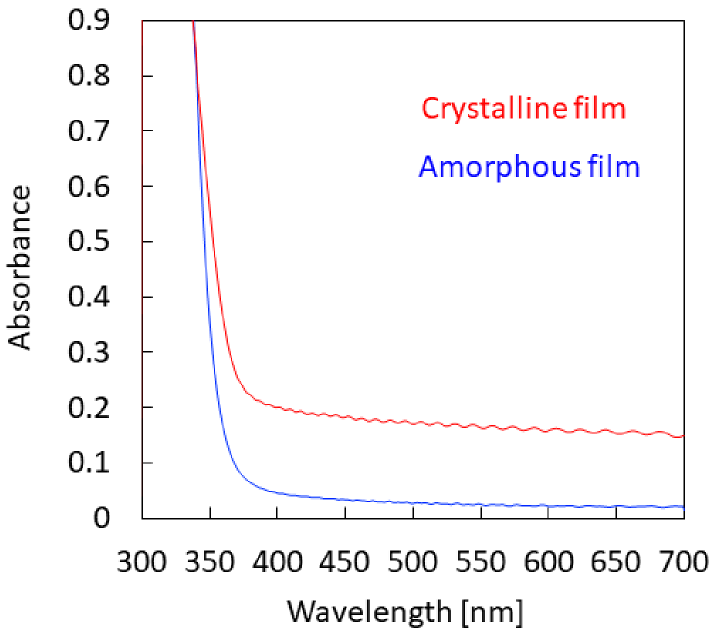

Figure S5. Absorbance changes before (blue) and after (red) crystallization of 10 wt% (*S*)-BINAP-doped (*S*)-H_8-BINAP films, which is measured by transmittance.

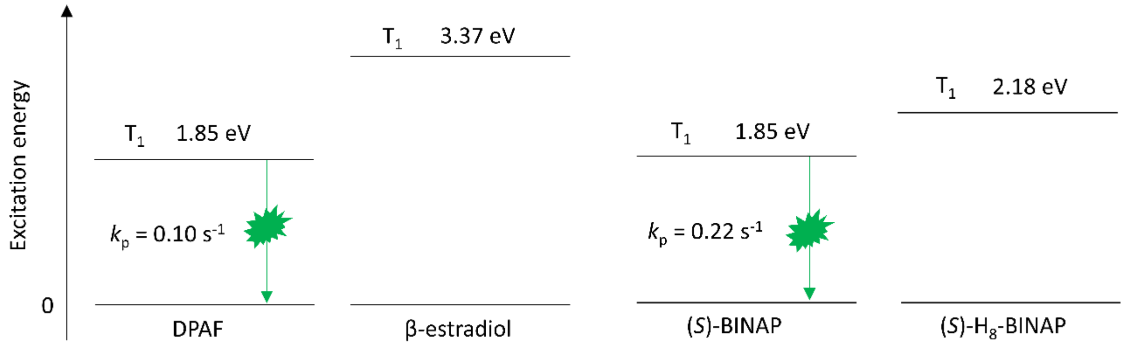

Figure S6. Calculated T_1 energy of DPAF, *β*-estradiol, (*S*)-BINAP, and (*S*)-H_8-BINAP. Optimized T_1 geometry was calculated using DFT (Gaussian09/B3LYP/6-31G(d)). T_1-S_0 energy is calculated using the ADF2018 software package with B3LYP Functional and PBE0 Basis set for the optimized T_1 geometries.

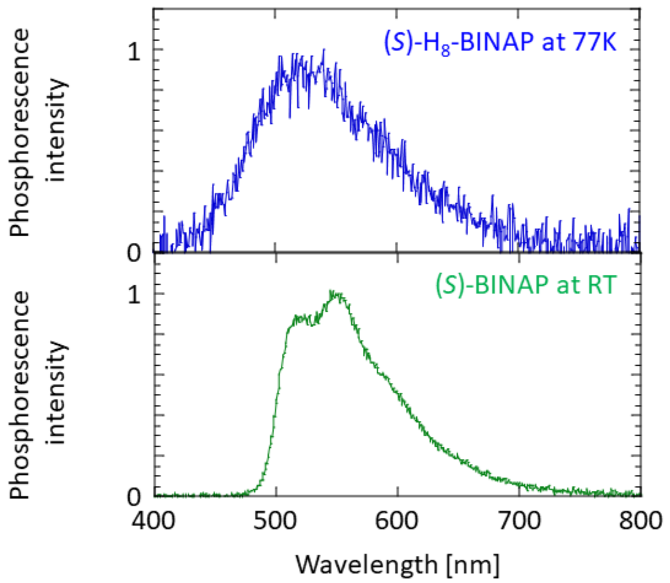

Figure S7. Phosphorescence spectra of (a) neat crystalline film of (*S*)-H_8-BINAP at 77 K and (b) 10 wt% (*S*)-BINAP doped in crystalline (*S*)-H_8-BINAP at RT.

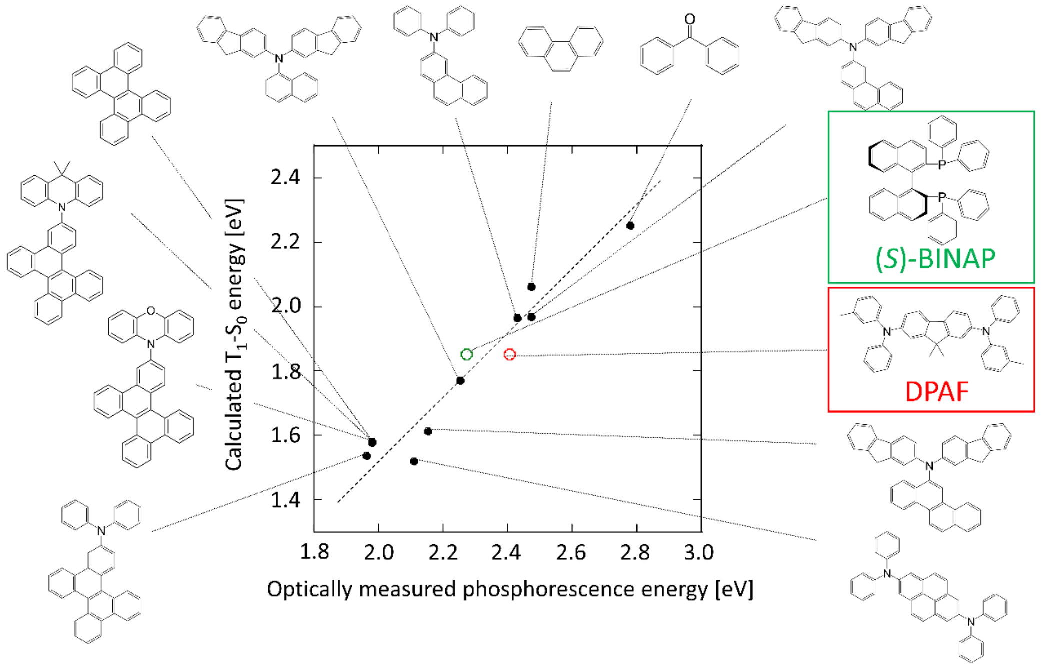

Figure S8. Relationship between the calculated T_1-S_0 energy (*y*-axis) and the peak wavelength of RTP (*x*-axis) for heavy-atom-free chromophores. Figure S20 in reference S5 shows data for the black plots.

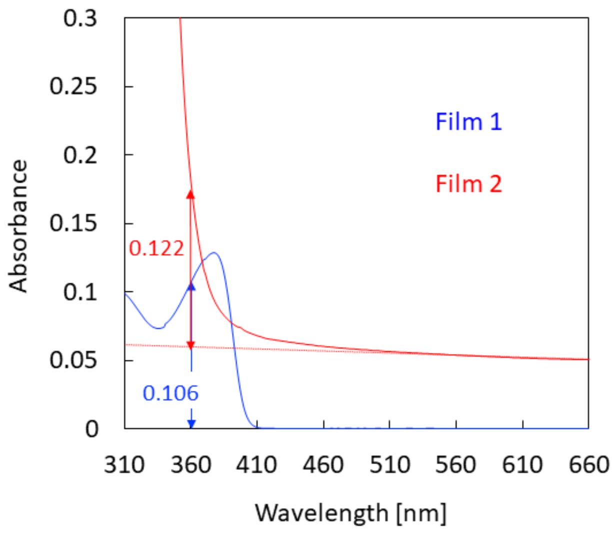

Figure S9. Absorption spectra of film 1 (1 wt% DPAF-doped amorphous *β*-estradiol film) and film 2 (10 wt% (*S*)-BINAP-doped crystalline (*S*)-H_8-BINAP film).

S5. Derivation of Equation 1

When the concentration of the ground state (S_0) is much larger than that of the lowest triplet excited state (T_1) and no exciton annihilation occurs except for triplet-triplet annihilation (TTA) caused by T_1 migration, the rate equation for the concentration of T_1 based on the conditions ($C_{T_1}{}'$) is expressed as:[S6,S7]

$$\frac{-dC_{T_1}{}'}{dt} = \frac{2.303\times10^3\varepsilon}{N_a} C_{S_0}\Phi_t I_{ex} - \frac{1}{2}k_{TT}C_{T_1}{}' - \frac{C_{T_1}{}'}{\tau_p(RT)}, \quad \text{(S1)}$$

where ε is the molar absorption coefficient in M^{-1} cm^{-1} at 360 nm, N_a is Avogadro's constant, C_{S_0} is the concentration of S_0 in M, Φ_t is the triplet generation yield, $\tau_p(RT)$ is the average lifetime of T_1 in s, k_{TT} is the rate constant of TTA, and I_{ex} is the excitation irradiance at 360 nm in photons s^{-1} cm^{-2}. In Equation S1, the first term represents the generation rate of T_1, the second term is the deactivation rate of T_1 by TTA, and the third term indicates the deactivation rate of T_1 from the T_1-S_0 transition. Because TTA deactivation was not observed in the data shown in the main manuscript, the second term is ignored to give the following rate equation:

$$\frac{-dC_{T_1}{}'}{dt} = \frac{2.303\times10^3\varepsilon}{N_a} C_{S_0}\Phi_t I_{ex} - \frac{C_{T_1}{}'}{\tau_p(RT)}, \quad \text{(S2)}$$

The solution of $C_{T_1}{}'$ in equation S2 is expressed as:

$$C_{T_1}{}' = \frac{2.303\times10^3\varepsilon}{N_a} C_{S_0}\Phi_t \tau_p(RT) I_{ex}, \quad \text{(S3)}$$

εC_{S_0} is commonly expressed using the Beer–Lambert law as:

$$\varepsilon C_{S_0} = \frac{Abs}{L}, \quad \text{(S4)}$$

where Abs is the absorbance at 360 nm, and L is the thickness of the film in cm. Combining Equation S3 and S4 results in the following form:

$$C_{\mathrm{T_1}}{}' = \frac{2.303\times10^3}{N_\mathrm{a}}\frac{\mathrm{Abs}}{L}\Phi_\mathrm{t}\tau_\mathrm{p}(\mathrm{RT})I_\mathrm{ex}. \quad \text{(S5)}$$

Equation S5 is usable in the range of excitation irradiance when the concentration of T_1 linearly increases with the elevated excitation irradiance. In real experimental results, the increase of the concentration of T_1 becomes saturated under strong excitation. The concentration of T_1 when it saturates at elevated excitation irradiance is defined as $C_{\mathrm{T_1}}$ which is expressed using the excitation intensity dependence of the RTP intensity $[E_\mathrm{p}(I_\mathrm{ex})]$ as:[S7,S8]

$$C_{\mathrm{T_1}} = \frac{E_\mathrm{p}(I_\mathrm{ex})}{E'_\mathrm{p}(I_\mathrm{ex})}C_{\mathrm{T_1}}{}', \quad \text{(S6)}$$

where $E'_\mathrm{p}(I_\mathrm{ex})$ is the RTP intensity based on the assumption that the saturation of the RTP intensity with elevated excitation irradiance does not occur. Equation S5 and S6 convert into Equation 1 used in the manuscript:

$$C_{\mathrm{T_1}} = \frac{E_\mathrm{p}(I_\mathrm{ex})}{E'_\mathrm{p}(I_\mathrm{ex})}\left[\frac{2.3\times10^3\mathrm{Abs}}{LN_\mathrm{a}}\right]\Phi_\mathrm{t}\tau_\mathrm{p}(\mathrm{RT})I_\mathrm{ex}. \quad (1)$$

S6. Estimation of triplet concentration by transient absorption

Because $\varepsilon_{\mathrm{T\text{-}T}}$ of DFAP is large, we were able to confirm the concentration of T_1 excitons with transient absorption measurements using the optical system of Figure S10a. Red plots in Figure S10b shows the relationship between ΔAbs at 650 nm and the excitation intensity at 360 nm for 1 wt% DFAP-doped amorphous *β*-estradiol film with a thickness of 2.0×10^{-4} cm. The red plots in Figure S10b can be represented by the following equation according to the Lambert–Beer law:

$$\Delta\mathrm{Abs}(\lambda) = \varepsilon_{\mathrm{T-T}}(\lambda)C_{\mathrm{T_1}}L, \quad \text{(S7)}$$

where L is the thickness of the solid film. When $C_{\mathrm{T_1}}$ at each excitation light intensity is calculated by substituting ΔAbs(650 nm) and the excitation light intensity into Equation S7, the relationship between $C_{\mathrm{T_1}}$ and the excitation intensity at 360 nm is obtained as shown in Figure S10c. Here, the value of $\varepsilon_{\mathrm{T\text{-}T}}$(650 nm) measured in benzene and $L = 2.0 \times 10^{-4}$ cm were used to determine $C_{\mathrm{T_1}}$ for each excitation intensity at 360 nm. The data

for the excitation light intensity dependences of C_{T_1} are equivalent to the red data in Figure 3b. Therefore, the similarity of the data in Figure S10c and Figure 3b indicates that C_{T_1} can be reasonably estimated from the *p*RTP intensity variation and Equation 1. Transient absorbance is difficult to measure at low excitation intensity. Therefore, determination of C_{T_1} using *p*RTP strength and Equation 1 appears to be more accurate. Furthermore, because of the low value of $\varepsilon_{T\text{-}T}$ of (*S*)-BINAP, C_{T_1} could not be determined from ΔAbs, and was instead determined from the *p*RTP intensity profile and Equation 1.

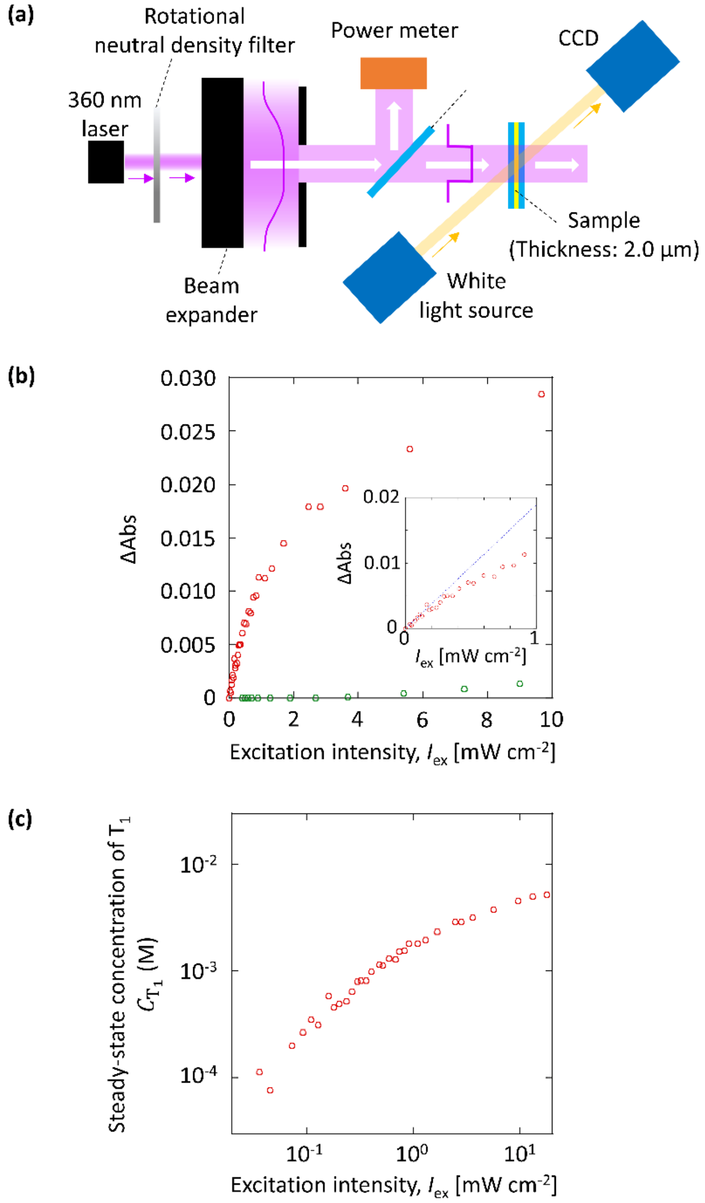

Figure S10. Determination of T_1 concentration using transient absorption. (a) An optical setup for transient absorption. (b) Relationship between ΔAbs and excitation irradiance

at 360 nm for film 1 (1 wt% DPAF-doped amorphous *β*-estradiol film; red plots) and film 2 (10 wt% (*S*)-BINAP-doped crystalline (*S*)-H_8-BINAP film; green plots). The blue line in inset graph represents a least squares straight line of the linear part of the data in Film 1. The absorbance of films 1 and 2 at 360 nm is 0.15 and 0.080, respectively. (c) The relationship between T_1 concentration and excitation irradiance at 360 nm for film 1, as determined from the data in (b).

S7. Consideration of maximum T_1 concentration at different dopant concentrations

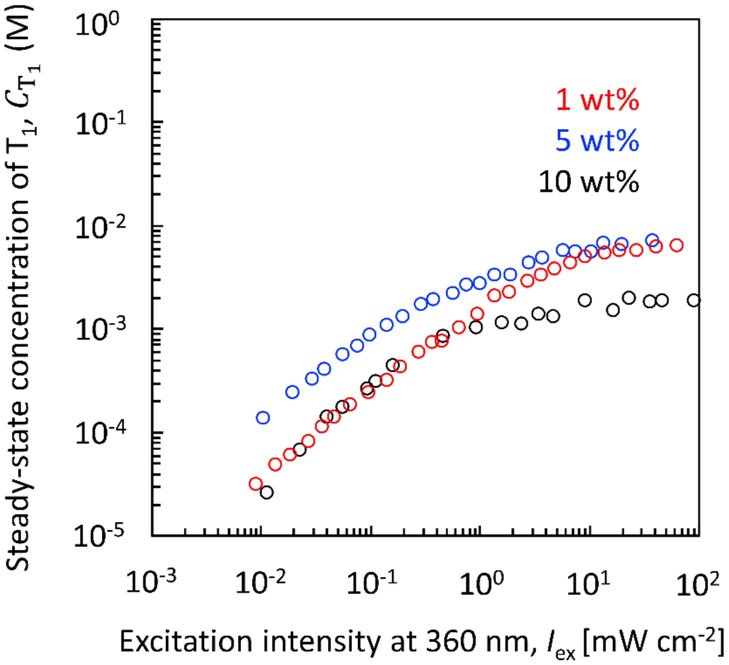

Figure S11. The relationship between C_{T_1} and excitation irradiance at 360 nm for 1.0 wt% DPAF-doped amorphous *β*-estradiol as film a (red), 5.0 wt% DPAF-doped amorphous *β*-estradiol as film b (blue), and 10 wt% DPAF-doped amorphous *β*-estradiol as film c (green). Absorbance at 360 nm of films a, b, and c are 0.093, 0.092, and 0.18, respectively.

Table S2. Summary of photophysical characteristics of 1.0 wt% DPAF-doped amorphous *β*-estradiol (film a) and 5.0 wt% DPAF-doped amorphous *β*-estradiol (film b).

Concentration [wt%]	Φ_p	τ_p(RT) [s]
1.0	0.063	1.19
5.0	0.048	1.02
10	0.0056	0.106

S8. Suppression of T_1 accumulation by triplet-triplet annihilation owing to triplet migration at high guest concentration

The reason for this decrease is that additional quenching by triplet-triplet annihilation (TTA) occurs. At low guest concentrations, quenching of $FRET_{S\text{-}T}$ or $FRET_{T\text{-}T}$ alone provides additional deactivation pathways when the excitation light intensity is high, because T_1 of the dye is constrained when the T_1 concentration of the dye is sufficiently lower than that of the host (Figure S12a). However, at high concentrations of the guest, T_1 migration and quenching by TTA occur (Figure S12b). In fact, in the sample of 10 wt% DFAP-doped amorphous *β*-estradiol, TTA-based upconversion fluorescence was weakly observed as an afterglow at high excitation light intensity (Figure S13). The two spectra in Figure S13 show the afterglow emission spectral intensities as the excitation light intensity was varied. The spectrum consists of blue afterglow fluorescence and green *p*RTP. Because the T_1 concentration is proportional to the *p*RTP intensity, the T_1 concentration increased to be 1.28 times as high as the excitation intensity from Figure S13a to Figure S13b. At this time the intensity of the blue afterglow fluorescence increased to 1.76, which is approximately the square of 1.28; hence, this blue afterglow fluorescence is likely to be TTA-based upconversion. Therefore, the formation of *π*-interacting states is not conducive to high T_1 concentrations. In 10 wt% (*S*)-BINAP-doped (*S*)-H_8-BINAP crystalline materials, no TTA-based upconversion fluorescence was observed at high excitation light intensity. (*S*)-BINAP is well-aligned in the crystal lattice of (*S*)-H_8-BINAP, and interactions between the two molecules are thought to be relatively small owing to the twisted structure. We have previously reported small electron and hole transfer integrals calculated for the crystal lattice of (*S*)-H_8-BINAP.[S9] Therefore, conditions that reduce the likelihood of quenching by TTA are also key to achieving a large T_1 accumulation.

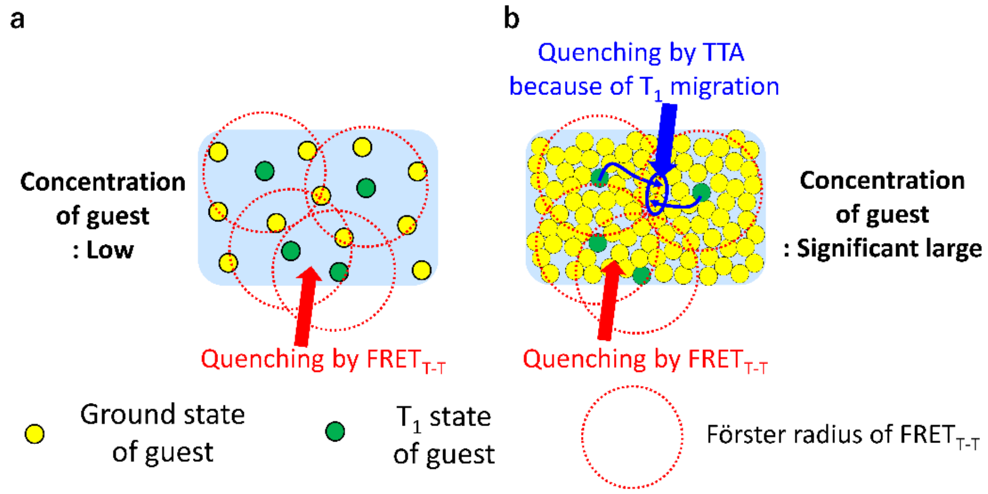

Figure S12. Differences in triplet accumulation behavior between low (a) and high (b) guest concentrations.

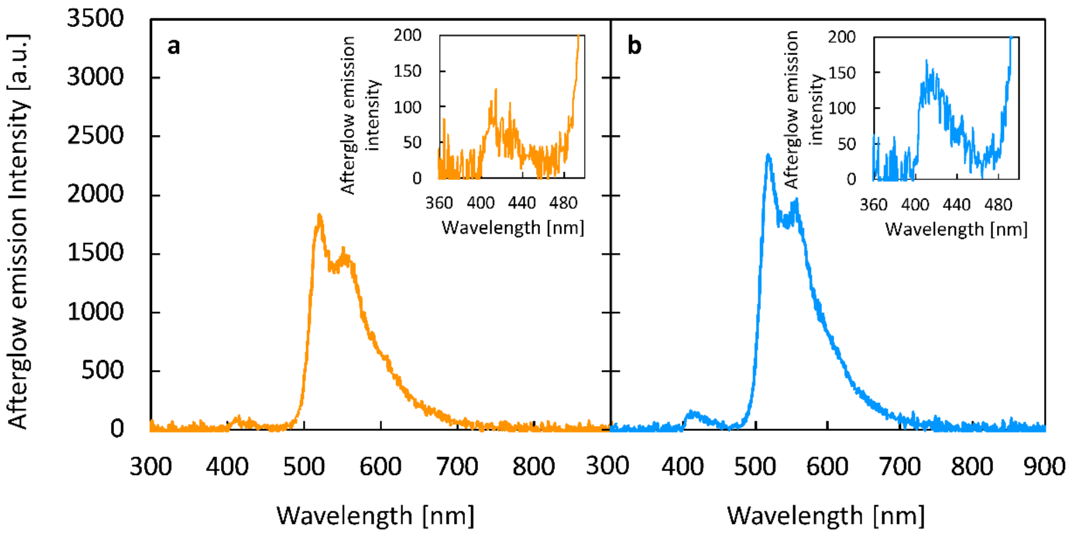

Figure S13. Afterglow emission spectral intensities of 10 wt% DFAP-doped amorphous β-estradiol films at different excitation intensities. (a) Excitation intensity is 8.0 mW cm^{-2}. (b) Excitation intensity is 16 mW cm^{-2}.

S9. Mandatory points to be checked to determine C_{T_1} using the excitation intensity dependence of RTP

The following three points (1)–(3) are must be considered to appropriately estimate C_{T_1} using the excitation intensity dependence of RTP, which has been partially reported in references S6 and S7.

(1) The absorbance value of the excitation wavelength is small (not near the magnitude of 1 or more,

but closer to 0.1 and 0.2).

(2) The absorbance should be measured using films without porous structures and pore sizes that are above the diffraction limit.

(3) Because of (1), estimation using a powder sample is not allowed.

Although bright persistent RT emission is crucial for high-resolution afterglow imaging for bioimaging and anticounterfeit applications, a nanomaterial with a size of less than 1 μm often has small absorbance because of the lower sample thickness. Because the brightness of the nanomaterials is proportional to T_1 concentration, the estimation of the upper limit regarding T_1 concentration for materials with a small absorbance at the excitation intensity is crucial. For example, we show the common relationship between afterglow RTP intensity and excitation irradiance for two films composed of the same material but with different absorbances, achieved by changing the thickness of the films, where one film has an absorbance of 0.1 at the excitation wavelength and the other film has an absorbance of 3 at the excitation wavelength (Figure S14). For the film with Abs = 0.1, the excitation intensity hardly changes along the depth of the film (Figure S14a). Because the profile of the T_1 concentration is almost the same along the depth direction of the film, the T_1 concentration homogeneously increases with excitation irradiance (blue → green → red → black in Figure S14a). Therefore, we can discuss the T_1 concentration using the increase of RTP intensity depending on elevated excitation irradiance from the films, as shown in Figure S14b and Figure 3a in the main text. However, the excitation intensity largely decreases along the depth direction for the film with Abs = 3 (Figure S14c), revealing a distribution of the T_1 concentration along the thickness of the sample. Under weak excitation, no saturation of T_1 concentration occurs along the depth direction (blue line in Figure S14c). With the increase in excitation power, the T_1 concentration ($\propto$ RTP intensity) in the surface region (Figure S14c (i)) becomes saturated with increasing excitation irradiance. However, the T_1 concentration ($\propto$ RTP intensity) in the deep region (Figure S14c (ii)) is not saturated compared with that in the surface region. We note the increased RTP intensity in the deep region does not include enough information to determine an upper limit of the T_1 concentration. Indeed, such large differences regarding the excitation intensity dependence of RTP intensity were observed for 1 wt% DPAF-doped amorphous *β*-estradiol film with different absorbances at 360 nm (Figure S15). Compared with 1 wt% DPAF-doped amorphous *β*-estradiol film with Abs = 0.17 at 360 nm (green plots in Figure S15), 1 wt% DPAF-doped amorphous *β*-estradiol film with Abs = 2 at 360 nm showed greater RTP intensity under stronger excitation (blue plots in Figure S15). Therefore, we overestimate the T_1 concentration when a film with large absorbance at the excitation wavelength is used because the

increase of RTP intensity under strong excitation irradiance occurs based on the scheme of Figure S15c. Overestimation of the T_1 concentration from the excitation intensity dependence of RTP intensity also occurs when a powder sample is used. This is because absorbance at the excitation wavelength often becomes large (approaching 1) when the excitation beam area is less than the area of the powder sample.

The overestimation of the T_1 concentration occurs when the film contains many holes with sizes greater than the diffraction limit. For the porous films, the absorbance value is greatly underestimated because significant irradiated light passes through the absence of material.

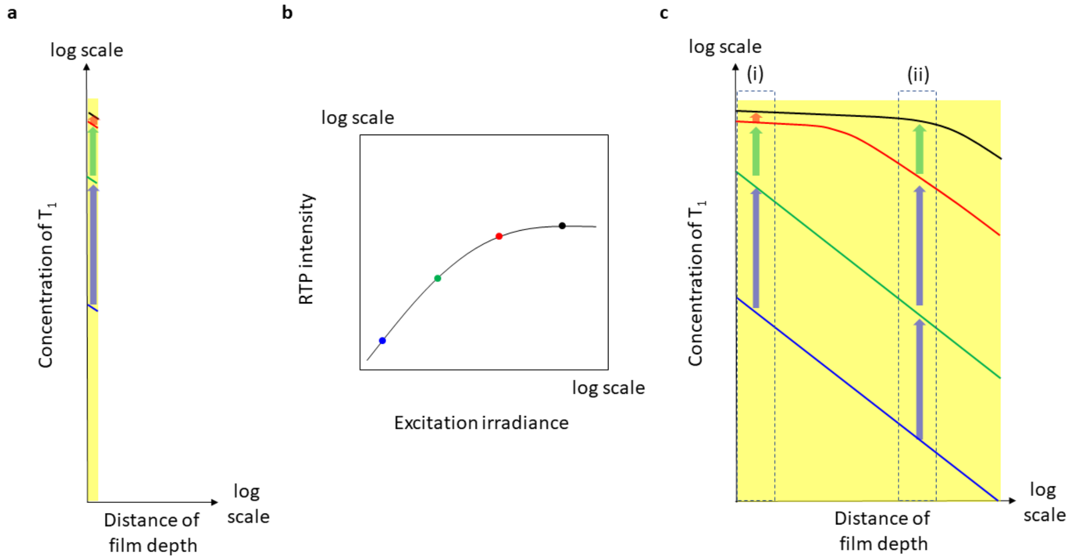

Figure S14. Change in the depth profile of RTP intensity (T_1 concentration) depending on different absorbances when excitation irradiance increases. (a) The T_1 concentration profile along the depth direction of the film when absorbance of the film at the excitation wavelength is 0.1. (b) The relationship between RTP intensity and excitation irradiance when absorbance of the film at the excitation wavelength is 0.1. (c) The T_1 concentration profile along the depth direction of the film when absorbance of the film at the excitation wavelength is 3.

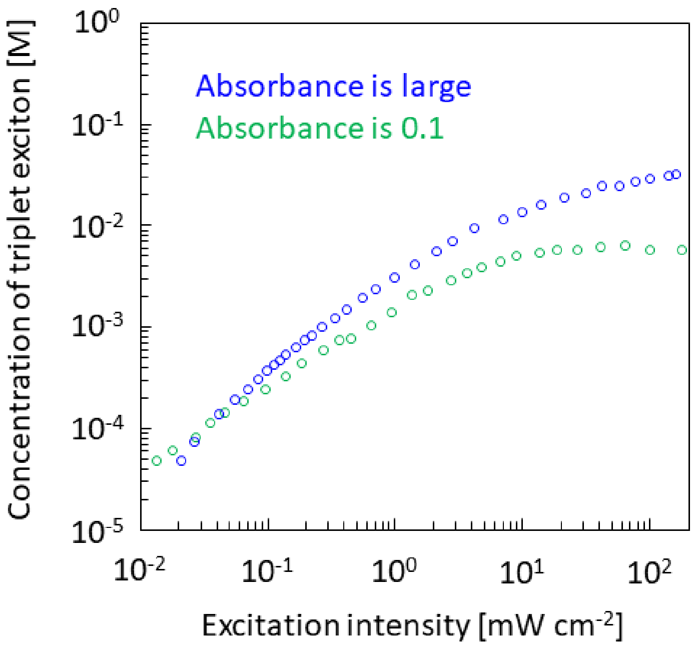

Figure S15. Different relationships between afterglow RTP intensity and excitation intensity at 360 nm for two 1 wt% DPAF-doped amorphous *β*-estradiol films with different absorbance values at the excitation wavelength.

S10. Measurement procedure of $\varepsilon_{\mathrm{T\text{-}T}}$

In transient absorption measurements, the Δabsorbance values of the sample and reference (ΔAbs^S and ΔAbs^R, respectively) are expressed as:

$$\Delta Abs^S = \alpha\varepsilon_{\mathrm{T\text{-}T}}{}^S\Phi_t{}^S Abs^S I_{ex}, \quad (S8)$$

$$\Delta Abs^R = \alpha\varepsilon_{\mathrm{T\text{-}T}}{}^R\Phi_t{}^R Abs^R I_{ex}, \quad (S9)$$

where $\varepsilon_{\mathrm{T\text{-}T}}{}^S$ and $\varepsilon_{\mathrm{T\text{-}T}}{}^R$ are the molar absorption coefficient of sample and reference, respectively, $\Phi_t{}^S$ and $\Phi_t{}^R$ are the triplet generation yield of sample and reference, respectively, Abs^S and Abs^R are the absorbances at the excitation wavelength of the sample and reference, respectively, α is a constant, and I_{ex} is the excitation irradiance. For the same absorbance at the excitation wavelength, the same I_{ex} and the same optical setup are used for the measurement, and then the following equation is obtained:

$$\varepsilon_{\mathrm{T\text{-}T}}{}^S = \varepsilon_{\mathrm{T\text{-}T}}{}^R[\Phi_t{}^R/\Phi_t{}^S]/[\Delta Abs^S/\Delta Abs^R]. \quad (S10)$$

In the experiment used to determine $\varepsilon_{T\text{-}T}$ of DPAF, benzophenone in benzene and DPAF in benzene were used as a reference and sample, respectively. The absorbance of two samples at 355 nm was set to 1.0. ΔAbs^S at 640 nm and ΔAbs^R at 532 nm under 355 nm excitation were measured (Figure S16). From Figure S16, $[\Delta Abs^S/\Delta Abs^R]$ is obtained. Φ_t^S of DPAF has been reported as 0.61 in benzene. Φ_t^R of benzophenone is known as 1. The $\varepsilon_{T\text{-}T}{}^R$ of benzophenone is reported as 7.6×10^3 $M^{-1}cm^{-1}$ at 532 nm in benzene. Therefore, using Equation S10, $\varepsilon_{T\text{-}T}$ at 640 nm for DPAF in benzene is determined to be 2.9×10^4 $M^{-1}cm^{-1}$.

To determine $\varepsilon_{T\text{-}T}$ of (*S*)-BINAP in tetrahydrofuran (THF), Φ_t^R of Bis(2,4-difluorophenylpyridinato)-tetrakis(1-pyrazolyl)borate iridium(III) (FIr6) in THF was initially determined. In the experiment, benzophenone in benzene and FIr6 in THF were used as a reference and sample, respectively. The absorbance of two samples at 355 nm was set to 1.0. ΔAbs^S at 440 nm and ΔAbs^R at 532 nm under 355 nm excitation were measured (Figure S17). Φ_t^R of benzophenone and Φ_t^S of FIr6 in THF are known as 1. The $\varepsilon_{T\text{-}T}{}^R$ of benzophenone is reported as 7.6×10^3 $M^{-1}cm^{-1}$ at 532 nm in benzene. $[\Delta Abs^S/\Delta Abs^R]$ is obtained from Figure S17. Therefore, using Equation S10, $\varepsilon_{T\text{-}T}$ for FIr6 in THF at 440 nm is determined to be 4.5×10^3 M^{-1} cm^{-1}.

Next, the data of $\varepsilon_{T\text{-}T}$ for FIr6 in THF at 440 nm was used to determine $\varepsilon_{T\text{-}T}$ of (*S*)-BINAP. In the experiment, FIr6 in THF and (*S*)-BINAP in THF were used as a reference and sample, respectively. The absorbance of two samples at 266 nm was set to 1.0. ΔAbs^S at 500 nm and ΔAbs^R at 440 nm under 266 nm excitation were measured (Figure S18). From Figure S18, $[\Delta Abs^S/\Delta Abs^R]$ is obtained. Φ_t^S of (*S*)-BINAP have been reported as 0.29 in THF. [S10] Φ_t^R of FIr6 is known as 1. The $\varepsilon_{T\text{-}T}{}^R$ of FIr6 is 4.5×10^3 $M^{-1}cm^{-1}$ at 440 nm in THF. Therefore, using Equation S8, $\varepsilon_{T\text{-}T}$ at 500 nm for (*S*)-BINAP in THF is determined to be 0.83×10^3 M^{-1} cm^{-1}. The data of $\varepsilon_{T\text{-}T}$ at 500 nm were used for the relationship between $\varepsilon_{T\text{-}T}$ and wavelength (Figure S18c), and Figure S18c was used in the bottom graphs of Figure 4b and 5b.

The $\varepsilon_{\mathrm{T-T}}$ values for DPAF dispersed in amorphous *β*-estradiol and (*S*)-BINAP dispersed in crystalline (*S*)-H_8-BINAP were measured using the following procedures. For the range of I_{ex} showing the linear relationship between $\Delta\mathrm{Abs}(\lambda)$ and $I_{\mathrm{ex}}(\lambda)$, the relationship between $\Delta\mathrm{Abs}(\lambda)$ is expressed using the Beer–Lambert law:

$$\Delta\mathrm{Abs}(\lambda) = \varepsilon_{\mathrm{T-T}}(\lambda) C_{\mathrm{T_1}}{}' L, \tag{S11}$$

The equation can be used for wavelengths where the S_0 of chromophores does not exist. From Equation S5 and S11, the following equation is obtained:

$$\Delta\mathrm{Abs}(\lambda) = \varepsilon_{\mathrm{T-T}}(\lambda)\frac{2.303\times10^{3}}{N_{\mathrm{a}}}\mathrm{Abs}\Phi_{\mathrm{t}}\tau_{\mathrm{p}}(\mathrm{RT})I_{\mathrm{ex}}, \qquad \text{(S12)}$$

For films 1 and 2, the relationships between $\Delta\mathrm{Abs}(\lambda)$ and I_{ex} were measured (Figure S10b). The linear part of inset in Figure S10b corresponds to a value of $\varepsilon_{\mathrm{T-T}}(\lambda)\frac{2.303\times10^{3}}{N_{\mathrm{a}}}\mathrm{Abs}\Phi_{\mathrm{t}}\tau_{\mathrm{p}}(\mathrm{RT})$ in Equation S12. By substituting Abs, Φ_t, and τ_p(RT) into the optically determined $\varepsilon_{\mathrm{T-T}}(\lambda)\frac{2.303\times10^{3}}{N_{\mathrm{a}}}\mathrm{Abs}\Phi_{\mathrm{t}}\tau_{\mathrm{p}}(\mathrm{RT})$, $\varepsilon_{\mathrm{T-T}}(\lambda)$ was determined. The $\varepsilon_{\mathrm{T-T}}(650)$ for DPAF doped in amorphous *β*-estradiol was 3.1 × 10^4 M^{-1} cm^{-1}, which was similar to results in benzene. The $\varepsilon_{\mathrm{T-T}}(\lambda)$ for (*S*)-BINAP in crystalline (*S*)-H_8-BINAP was difficult to accurately determine because of the noise level, even when strong excitation light was used (Figure S10b). At least, the $\varepsilon_{\mathrm{T-T}}$ for (*S*)-H_8-BINAP in crystalline (*S*)-H_8-BINAP (film 2) was less than that in THF for the whole range of visible wavelengths. Therefore, $\varepsilon_{\mathrm{T-T}}$ for (*S*)-H_8-BINAP in THF (Figure S18c) is considered an upper limit of $\varepsilon_{\mathrm{T-T}}$ regarding (*S*)-H_8-BINAP in film 2.

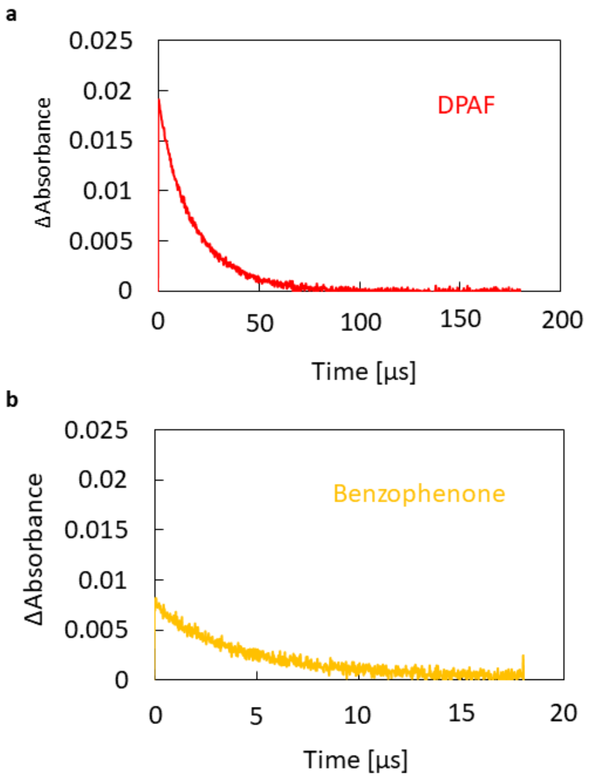

Figure S16. Transient absorption decay characteristics of (a) DPAF in degassed benzene at 640 nm and (b) benzophenone in degassed benzene at 532 nm.

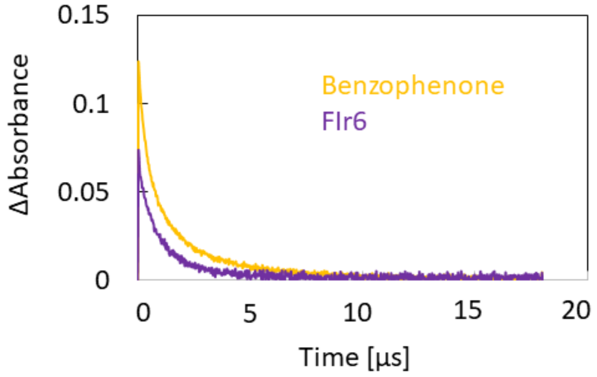

Figure S17. Transient absorption decay characteristics of FIr6 in degassed THF at 440 nm and benzophenone in degassed benzene at 532 nm.

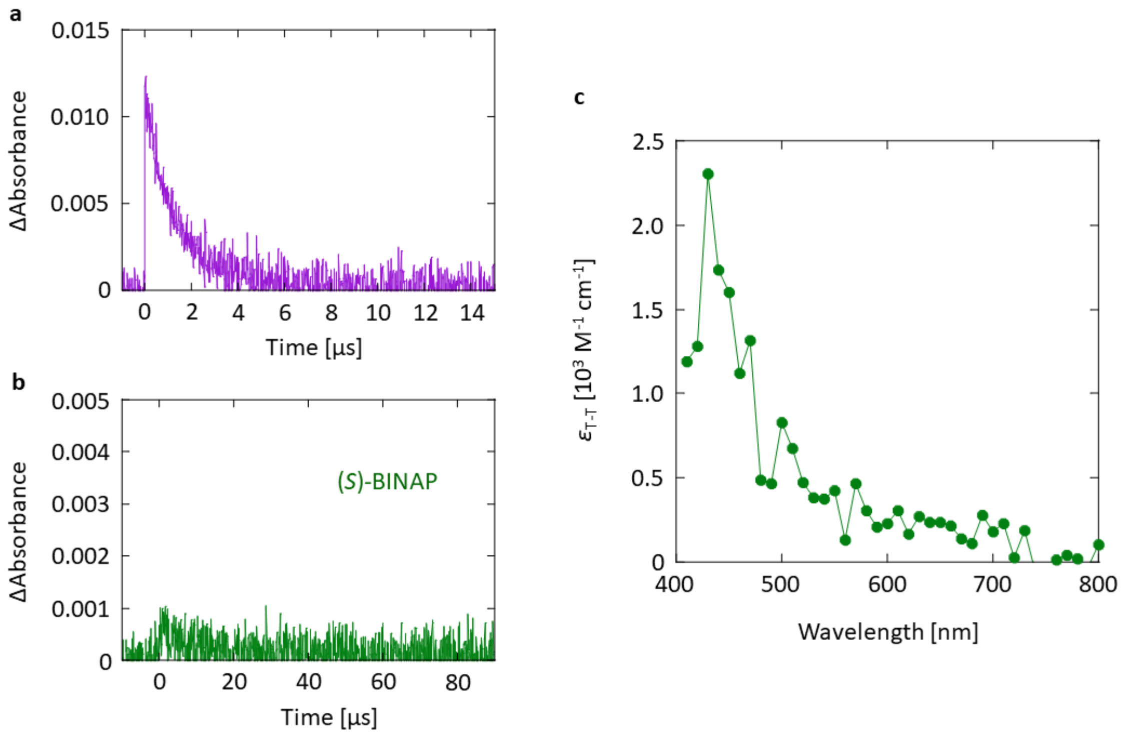

Figure S18. (a) Transient absorption decay characteristics of FIr6 in degassed THF at 440 nm and (b) (*S*)-BINAP in degassed THF. (c) T-T absorption spectra of (*S*)-BINAP in THF at 500 nm.

S11. Quantum chemical calculation of $\varepsilon_{T\text{-}T}$

The T-T absorption spectra and magnitude of $\varepsilon_{T\text{-}T}$ for dispersed conjugated chromophores have been well calculated using DFT.[S7,S11] Therefore, the T-T absorption characteristics of DPAF and (*S*)-BINAP can be calculated. DPAF has a large oscillator strength for the T_1-T_{12} transition at 618 nm (top in Figure S19a, top). This corresponds to the optically observed large $\varepsilon_{T\text{-}T}$ of DPAF with an approximate peak wavelength of 650 nm. The T_1-T_{12} transition is composed of the electron transfer between the HOMO and LUMO+6, and the overlapping density of the two orbitals is delocalized for the whole DPAF (Figure S19b, left). Because the distance of the overlap density along the *x*-axis is long, the large oscillator strength for the T_1-T_{12} transition is observed. However, the calculation regarding T-T absorption of (*S*)-BINAP indicates that no transitions with large oscillator strengths exist in the visible wavelengths (Figure S19a, bottom). Although many transitions exist for wavelengths of more than 420 nm, all of the oscillator strengths of the transitions are less than 0.01. Because most of the transitions have charge transfer (CT) characteristics, the small oscillator strength may be explained by a large separation of orbitals relating to the transition. For the T-T absorption at wavelengths less than 420 nm, the oscillator strength of the T_1-T_{28} transition is 0.07 (Figure S19a, bottom), which is moderate but not large. The moderate oscillator strength of the T_1-T_{28} transition corresponds to the (*S*)-BINAP that has $\varepsilon_{T\text{-}T}$ with a magnitude of 10^3 cm^{-1} M^{-1} at approximately 400 nm. This explains the values of optically observed $\varepsilon_{T\text{-}T}$ of (*S*)-BINAP at 410–470 nm. The T_1-T_{28} transition is composed of the transition between the HOMO and LUMO+11 and the transition between HOMO-13 and LUMO (Figure S15b, right). Because the transition between the HOMO-13 and LUMO has a strong CT character, it hardly enhances $\varepsilon_{T\text{-}T}$. Although the transition between the HOMO and LUMO+11 has significant overlap, a symmetry-forbidden transition dipole along the *y*-axis suppresses $\varepsilon_{T\text{-}T}$. Thus, transitions with many CT characteristics, as well as the symmetric forbidden local excited state characteristics, explain the significantly small $\varepsilon_{T\text{-}T}$ for the whole range of visible wavelengths, contributing to the suppression of the triplet depletion of $FRET_{S\text{-}T}$ and $FRET_{T\text{-}T}$ under strong excitation.

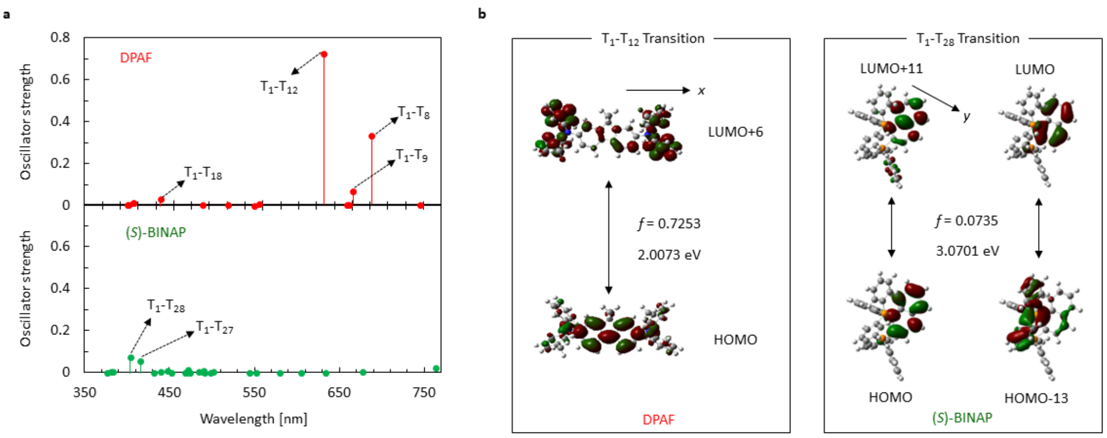

Figure S19. Analysis of small $\varepsilon_{\text{T-T}}$ for the visible wavelengths using quantum chemical calculation. (a) Calculated oscillator strength of T-T absorption for DPAF (top) and (*S*)-BINAP (bottom). (b) Description of representative molecular orbitals related to the T-T transition with large oscillator strengths (*f*) of DPAF (left) and (*S*)-BINAP (right).

S12. Potential mechanism by which FRET$_{\text{T-S}}$ lowers triplet generation yield

Saturation behavior of fluorescence for DPAF in Figure 4a can be considered as deactivation caused by FRET$_{\text{S-T}}$ for the following reasons: when the excitation is weak, few T_1 excitons exist near S_1 states formed by excitation. In this situation, S_1 excitons contribute to fluorescence and T_1 generation via intersystem crossing (ISC) from S_1 because internal conversion from S_1 can be ignored for the chromophores in this manuscript. The fluorescence yield (Φ_{f}') under weak excitation can be expressed as:

$$\Phi_{\text{f}}' = k_{\text{f}}/(k_{\text{f}} + k_{\text{isc}}), \qquad \text{(S12)}$$

where k_{f} is the fluorescence rate constant from S_1 and k_{isc} is the rate constant of ISC from S_1. However, an additional S_1 quenching pathway, FRET$_{\text{S-T}}$, occurs because the concentration of T_1 increases under strong excitation. When we define the rate constant of FRET$_{\text{S-T}}$ as $k_{\text{FRET T-S}}$, Φ_{f}' under strong excitation (Φ_{f}) can be expressed as:

$$\Phi_{\text{f}} = k_{\text{f}}/(k_{\text{f}} + k_{\text{isc}} + k_{\text{FRET S-T}}). \qquad \text{(S13)}$$

Therefore, Φ_{f} decreases when FRET$_{\text{S-T}}$ occurs under strong excitation. A lower fluorescence yield was observed at saturation of the fluorescence at high excitation intensity, as shown in Figure 4a. Hence, the ratio of $\Phi_{\text{f}} / \Phi_{\text{f}}'$ corresponds to $E_{\text{p}}(I_{\text{ex}})/E_{\text{p}}'(I_{\text{ex}})$ in Figure 4a [$\Phi_{\text{f}}/\Phi_{\text{f}}' = E_{\text{p}}(I_{\text{ex}})/E_{\text{p}}'(I_{\text{ex}})$]

Next, we note that $E_{\mathrm{p}}(I_{\mathrm{ex}})/E'_{\mathrm{p}}(I_{\mathrm{ex}})$, in Figure 4a, could be used to quantitatively estimate the decrease of the T_1 generation yield, which under weak excitation ($\Phi_{\mathrm{isc}}{}'$) can be expressed as:

$$\Phi_{\mathrm{isc}}{}' = k_{\mathrm{f}}/(k_{\mathrm{f}} + k_{\mathrm{isc}}). \quad \text{(S14)}$$

Because the term $k_{\mathrm{FRET\ S\text{-}T}}$ contributes under strong excitation, the T_1 generation yield under weak excitation (Φ_{isc}) can be expressed as:

$$\Phi_{\mathrm{isc}} = k_{\mathrm{f}}/(k_{\mathrm{f}} + k_{\mathrm{isc}} + k_{\mathrm{FRET\ S-T}}). \quad \text{(S15)}$$

From equations (S12)–(S15), we obtain the following relationship:

$$E_{\mathrm{p}}(I_{\mathrm{ex}})/E'_{\mathrm{p}}(I_{\mathrm{ex}}) = \Phi_{\mathrm{f}}/\Phi_{\mathrm{f}}{}' = \Phi_{\mathrm{isc}}/\Phi_{\mathrm{isc}}{}'. \quad \text{(S16)}$$

Therefore, data for $E_{\mathrm{p}}(I_{\mathrm{ex}})/E'_{\mathrm{p}}(I_{\mathrm{ex}})$ shown in Figure 4a can be used to quantitatively estimate the magnitude of the decrease in T_1 generation yield under weak and strong excitation.

S13. Calculation of *d*

The average distance between two triplet states (*d*) was determined based on T_1 distributed via hexagonal close-packing. *d* was approximated using:[S12]

$$24\sqrt{2}r^3 = \frac{1}{6\times1000N_{\mathrm{a}}c_{\mathrm{T_1}}}, \quad \text{(S17)}$$
$$d = 2r. \quad \text{(S18)}$$

When the concentration of the lowest singlet excited state (S_1) is much smaller than the continuously accumulated T_1 under continuous excitation, d is also considered as the distance between S_1 and T_1 under excitation. This estimation is appropriate because the S_1 lifetime is much shorter than the T_1 lifetime and no transient absorption caused by S_1 is observed in the power range of continuous excitation in this paper.

S14. Effect of photoinduced ionization under strong excitation irradiance

In recent years, triplet depletion via photoinduced ionization (PII) has been gaining attention.[S13-S15] Recent detailed analysis explains that T_1 is consumed when electrons reach the vacuum state from the LUMO of a guest chromophore under strong excitation (Figure S16a), and the electron mostly goes back to the guest chromophore to form S_1

and rapidly form S_0 to depopulate T_1.[S16] As proof that the PII also affects the saturation of C_{T_1} of film 2 under very large excitation intensity, the afterglow RTP image depleted by strong light irradiation at 445 nm, which does not contribute to S_0-S_1 excitation of (S)-BINAP, is irradiated (Figure S16b and S16c). If decrease of E_p/E_f with elevated excitation intensity (Figure 5a) is caused by $FRET_{T\text{-}T}$, E_f of film 2 should also be saturated with increasing excitation irradiance for film 2 because $R_{S\text{-}T}$ and $R_{T\text{-}T}$ are not sufficiently different. However, such distinct saturation was selectively observed for phosphorescence in film 2 (Figure 5a). This also supports PII via T_1 (Figure S16a) becoming a main triplet quenching pathway for excitation intensity greater than 30 mW cm^{-2} for film 2. However, the triplet saturation caused by $FRET_{S\text{-}T}$ and $FRET_{T\text{-}T}$ under strong excitation is greatly suppressed because of the significantly small $\varepsilon_{T\text{-}T}$, and this allows the continuous C_{T_1} accumulation of more than 1 wt%. Therefore, this report also quantitatively clarifies an additional point, demonstrating that the exploration of chromophores with small $\varepsilon_{T\text{-}T}$ is necessary for brighter afterglow emission. The suppression of the PII via T_1 is challenging but necessary to enhance C_{T_1} and achieve brighter afterglow RTP.

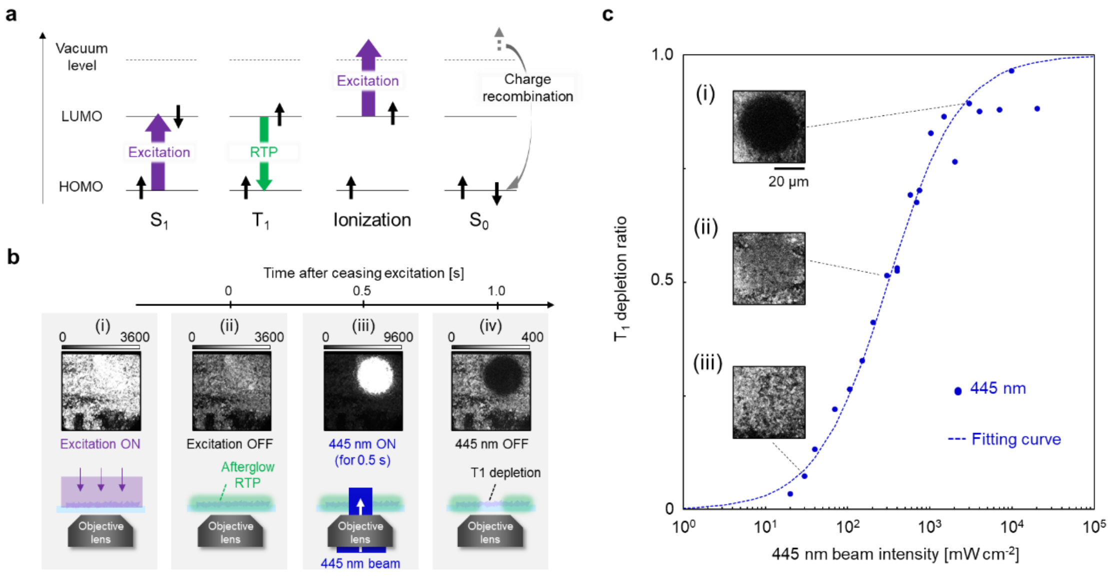

Figure S20. Effect of photoinduced ionization in film 2. (a) Energy diagrams illustrating afterglow RTP depletion due to the photoinduced ionization (PII) under strong excitation irradiance. (b) Microscopic emission images of film 2 (i) under a 360 nm excitation irradiance, (ii) soon after the excitation ceased, (iii) under irradiation with a 445 nm beam,

and (iv) soon after the 445 nm beam ceased. The intensities of the 360 nm excitation and the 445 nm beam were 1.0 mW cm^{-2} and 9.9 W cm^{-2}, respectively. The 445 nm beam irradiation was for 0.5 s. (c) Relationship between the triplet depletion ratio and the 445 nm beam intensity (I). The triplet depletion ratio was determined by comparison of afterglow RTP intensity in the absence and presence of the 445 nm beam irradiance. A dashed line is a fitting curve with model $1/(1 + I_{0.5}/I)$, where $I_{0.5}$ is the 445 nm beam intensity at which the afterglow RTP intensity becomes half its initial value. The 445 nm beam irradiation was for 0.5 s. Insert photographs (i), (ii), and (iii) show persistent RTP images soon after ceasing the 445 nm beam at powers of 3.0×10^{0}, 3.0×10^{-1}, and 3.0×10^{-2} W cm^{-2}, respectively.

S15. Theoretical RTP brightness of individual particles with sizes below the diffraction limit

In microscope epi-emission imaging, the total RTP intensity observed from an individual particle (F_p) could be expressed using the total number of T_1 in the particle as:

$$F_p = \beta \tau_p(\mathrm{RT})^{-1} \Phi_r^{T}(\mathrm{RT}) N_{T_1}, \qquad \text{(S19)}$$

where β is the constant number determined by optics and sensitivity of the photodetector, τ_p(RT) is the T_1 lifetime at RT, Φ_r^T(RT) is the radiation yield from T_1 at RT without including the triplet generation yield from S_1, and N_{T_1} is the number of triplet excition in the particle. $\tau_p(\mathrm{RT})^{-1}\Phi_r^{T}(\mathrm{RT})N_{T_1}$ corresponds to phosphorescence photon numbers per 1 s generated by the particle. Equation S19 indicates no observation of RTP from materials with zero Φ_r^T(RT) even when large numbers of T_1 exist in the particle. τ_p(RT) and Φ_r^T(RT) are commonly expressed as:

$$\Phi_r^{T}(\mathrm{RT}) = k_p/(k_p + k_{nr} + k_q), \qquad \text{(S20)}$$

$$\tau_p(\mathrm{RT})^{-1} = (k_p + k_{nr} + k_q), \qquad \text{(S21)}$$

where k_p is the rate constant of phosphorescence, k_{nr} is the rate constant of radiationless transition from T_1 depending on the intramolecular process of chromophores, k_q is the triplet quenching rate of the guest chromophore caused by intermolecular interactions between the guest and the host. Because N_{T_1} is a multiplication of the concentration of T_1 (C_{T_1}) and the volume of the individual particle (V), Equation S19–S21 convert into:

$$F_p = \beta k_p C_{T_1} V. \qquad \text{(S22)}$$

When the afterglow RTP brightness of two different materials with the same V, the comparison of $k_p C_{T_1}$ is crucial because β is constant, independent of the material. The upper limitation of $k_p C_{T_1}$ of solid B is approximately 10 times compared with that of solid A; solid B intrinsically has great afterglow intensity capability.

S16. Transmission electron microscope images of individual crystals

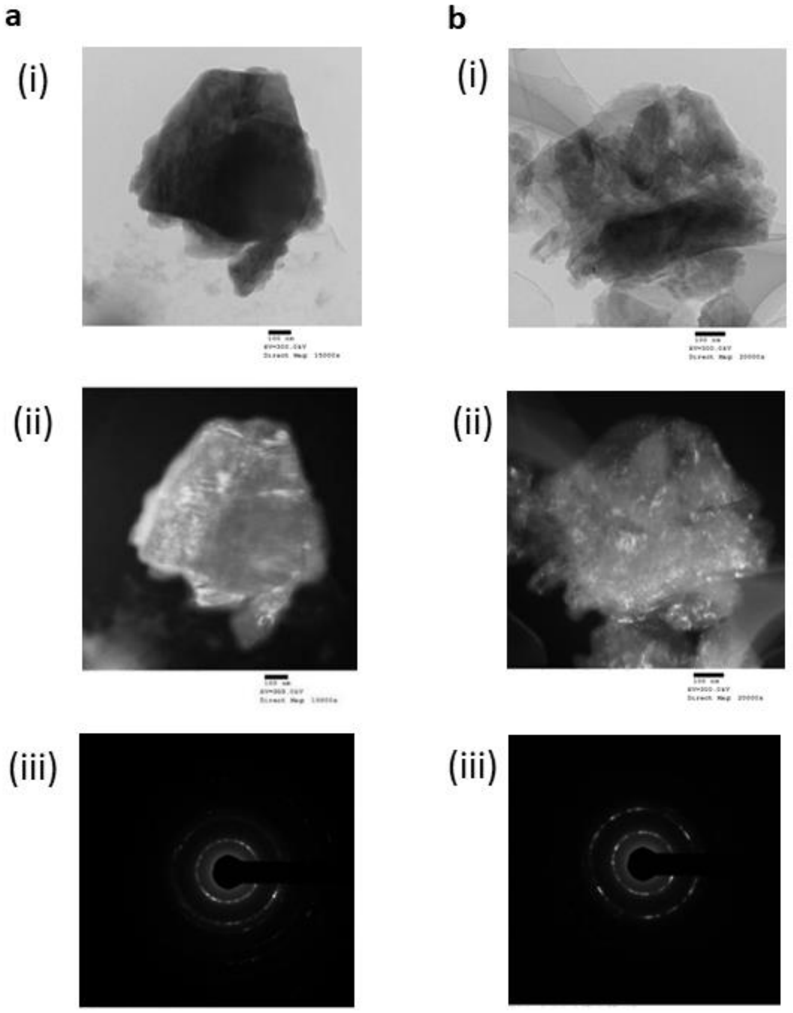

Figure S21. TEM images [(i) bright field and (ii) dark field images] and (iii) diffraction pattern of individual particles. (a) 10 wt% (*S*)-BINAP-doped (*S*)-H_8-BINAP crystals. (b) 1 wt% DPAF-doped (*S*)-H_8-BINAP crystals. In (i) and (ii), scale bar is 100 nm.

S17. Maximum triplet concentration when the dopant concentration is small

The relationship between C_{T_1} and excitation intensity using 0.1 wt% (*S*)-BINAP-doped (*S*)-H_8-BINAP crystalline films was estimated using the same procedure as explained in the main manuscript. Initially, the relationship between the RTP intensity and 360 nm excitation irradiance of the 0.1 wt% (*S*)-BINAP-doped (*S*)-H_8-BINAP crystalline film with the thickness (L) of 10 µm was measured (Figure S22a). The Φ_p(RT) and τ_p(RT) of the 0.1 wt% (*S*)-BINAP-doped (*S*)-H_8-BINAP crystalline film were 0.0022 and 0.44 s, respectively. By substituting Φ_p(RT), τ_p(RT), and $k_p = 0.25\ s^{-1}$ into $\Phi_p(RT) = \Phi_t k_p \tau_p(RT)$, Φ_t was determined to be 0.020. Absorbance at 360 nm (Abs) of the 0.1 wt% (*S*)-BINAP-doped (*S*)-H_8-BINAP crystalline film was 0.052. By substituting data from Figure S22a for $[\frac{E_p(I_{ex})}{E'_p(I_{ex})}]$ and $\left[\frac{2.3\times10^3\text{Abs}}{LN_a}\right]\Phi_t\tau_p(\text{RT})$ into Equation 1, the relationship between C_{T_1} and I_{ex} of the 0.1 wt% (S)-BINAP-doped (*S*)-H_8-BINAP crystalline film was obtained (blue plots in Figure S22b).

For the 10 wt% (*S*)-BINAP-doped (*S*)-H_8-BINAP crystalline film, C_{T_1} increases up to 2.3×10^{-2} M (green in Figure S22b). However, C_{T_1} does not increase up to 1×10^{-3} M for 0.1 wt% (*S*)-BINAP-doped (*S*)-H_8-BINAP crystalline films, even when the excitation irradiance increases up to 10 W cm^{-2} (blue in Figure S22b). When the S_0 concentration of the RTP center is approximately 1×10^{-3} M for 0.1 wt% (*S*)-BINAP-doped (*S*)-H_8-BINAP crystalline films, C_{T_1} cannot increase more than 1×10^{-3} M because the RTP center is continuously filled under strong excitation. Indeed, the brightness of green persistent RTP for 0.1 wt% (*S*)-BINAP-doped (*S*)-H_8-BINAP crystalline films (Figure S22b (ii)) is weak compared with that of 10 wt% (*S*)-BINAP-doped (*S*)-H_8-BINAP crystalline films (Figure S22b (i)) in the 360 nm irradiance of 200 mW cm^{-2}. The brightness of 0.1 wt% (*S*)-BINAP-doped (*S*)-H_8-BINAP crystalline films does not increase for 360 nm irradiance of approximately 5.0 W cm^{-2} (Figure S22b (iii)). If the concentration of the RTP center is potentially unclear because of the very small concentration of the impurities, it may be estimated using the procedure used in this manuscript. If C_{T_1} does not increase significantly, there is a possibility that the highly concentrated aggregates of the conjugated molecular crystals do not cause the RTP, but low concentrated impurities with small T_1 energy act as a triplet trap as the RTP center.

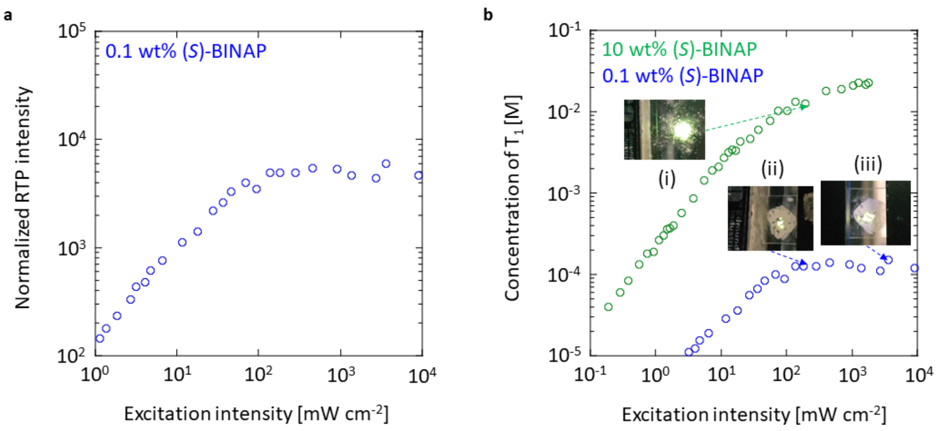

Figure S22. Afterglow RTP intensity and triplet concentration depending on excitation intensity of the 0.1 wt% (S)-BINAP-doped (*S*)-H_8-BINAP crystalline film (blue) and 10 wt% (S)-BINAP-doped (*S*)-H_8-BINAP crystalline film (red). (a) The relationship between afterglow RTP intensity and excitation intensity at 360 nm. (b) The relationship between continuously accumulated triplet concentration and excitation intensity at 360 nm. Inset (i)-(iii) represent photos showing persistent RTP brightness from samples soon after ceasing excitation under room light.

S18. Supporting movies

In Movie S1 and S2, an electron multiplication CCD camera (iXon Ultra/life 897, Andor Technology, United Kingdom) was used to capture the movies as tiff files. The CCD was cooled to −80 °C. In Movie S1 and S2, the setting condition regarding maximum brightness and minimum brightness of the tiff files was changed using ImageJ to appropriately compare the differences of each sample, as summarized in Table S3. In Table S3, the setting of the maximum brightness corresponds to the maximum intensity of the first two-dimensional images after excitation completely ceased. The setting of the minimum brightness corresponds to the background intensity caused by the heat-driven current. The tiff files were converted to AVI format using ImageJ without changing video parameters.

Movie S1. A movie of afterglow images from an anticounterfeit media used in Figure 1d soon after excitation at 360 nm ceased. The intensity of the 360 nm beam is 0.1 mW cm^{-2}. The size of the images is 400 μm × 400 μm. Images were used for (i) in Figure 1d.

Movie S2. A movie of afterglow images from an anticounterfeit media used in Figure 1d soon after excitation at 360 nm ceased. The intensity of the 360 nm beam is 300 mW cm^{-2}. The size of the images is 400 μm × 400 μm. Images were used for (v) in Figure 1d.

Table S3. Setting condition of CCD for taking movies.

Excitation power density ($mW\ cm^{-2}$)	Setting of minimum brightness	Setting of maximum brightness	Exposure time s	Gain
0.1	307	32582	0.5	1000
300	454	1899	0.5	1

S19. Supporting references

[S1] X. Zhen, Y. Tao, Z. An, P. Chen, C. Xu, R. Chen, W. Huang, K. Pu, *Adv. Mater.* **2017**, *29*, 1606665.

[S2] O. V. Dolomanov, L. J. Bourhis, R. J. Gildea, J. A. K. Howard, H. Puschmann, *J. Appl. Cryst.* **2009**, *42*, 339–341.

[S3] H. Nakanotani, M. Saito, H. Nakamura, C. *Adv. Funct. Mater.* **2010**, *20*, 1610–1615.

[S4] C. Chen, Z. Chi, K. C. Chong, A. S. Batsanov, Z. Yang, Z. Mao, Z. Yang, B. Liu. *Nat. Mater.* **2021**, *20*, 175–180.

[S5] K. Fukasawa, Y. Sugawara, R. Tsuru, T. Yamashita, S. Hirata, *J. Phys. Chem. Lett.* **2022**, *13*, 7788–7796.

[S6] M. A. Baldo, C. Adachi, and S. R. Forrest, *Phys. Rev. B* **2000**, 62, 10967.

[S7] S. Hirata, K. Totani, T. Yamashita, C. Adachi, M. Vacha, *Nat. Mater.* **2014**, *13*, 938–946.

[S8] S. Hirata, M. Vacha, *Adv. Opt. Mater.* **2016**, *4*, 297–305.

[S9] S. Hirata, H. Hara, I. Bhattacharjee, *J. Phys. Chem. C* **2020**, *124*, 25121–25132.

[S10] T. Kusama, S. Hirata, *Front. Chem.* **2021**, *9*, 788577.

[S11] S. Hirata, M. Vacha, *J. Phys. Chem. Lett.* **2016**, *7*, 1539–1545.

[S12] B. Sk, R. Tsuru, K. Hayashi, S. Hirata, *Adv. Funct. Mater.* **2023**, *33*, 2211604.

[S13] H. Ohkita, W. Sakai, A. Tsuchida, T. Yamamoto, *Macromolecules* **1997**, *30*, 5376–5383.

[S14] W. Li, Z. Li, C. Si, M. Y. Wong, K. Jinnai, A. K. Gupta, R. Kabe, C. Adachi, W. Huang, E. Zysman-Colman, I. D. W. Samuel, *Adv. Mater.* **2020**, *32*, 2003911.

[S15] X. Liang, Y. X. Zheng, J. L. Zuo, *Angew. Chem. Int. Ed.* **2021**, *60*, 16984–16988.

[S16] K. Hayashi, K. Fukumoto, S. Hirata, *ACS Mater. Lett.* **2023**, *5*, 1649–1655.
